# A High-Throughput Integrated Nontargeted Metabolomics and Lipidomics Workflow Using Microelution Enhanced Matrix Removal-Lipid for Comparative Analysis of Human Maternal and Umbilical Cord Blood Metabolomes

**DOI:** 10.1021/acs.analchem.4c03222

**Published:** 2025-01-30

**Authors:** Wenjie Wu, Ke Wang, Jianing Liu, Pui-Kin So, Ting-Fan Leung, Man-sau Wong, Danyue Zhao

**Affiliations:** a Department of Food Science and Nutrition, The Hong Kong Polytechnic University, Hong Kong 999077, China; b Centre for Eye and Vision Research (CEVR), 17W Hong Kong Science Park, Hong Kong China; c University Research Facility in Life Sciences, The Hong Kong Polytechnic University, Hong Kong 999077, China; d Department of Paediatrics, The Chinese University of Hong Kong, Prince of Wales Hospital, Shatin, Hong Kong SAR China; e Hong Kong Hub of Paediatric Excellence, The Chinese University of Hong Kong, Shatin, Hong Kong SAR China; f Research Institute for Future Food, The Hong Kong Polytechnic University, Hong Kong 999077, China; g Research Center for Chinese Medicine Innovation, The Hong Kong Polytechnic University, Hong Kong 999077, China

## Abstract

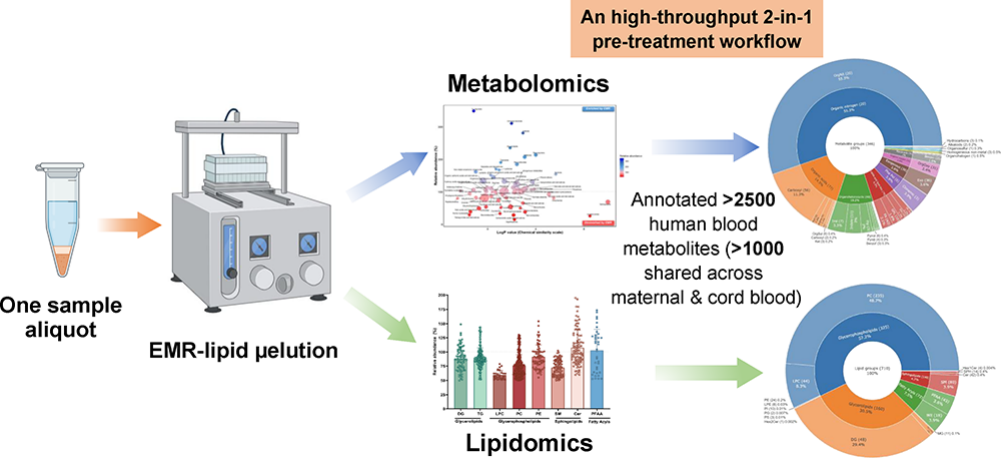

Sample pretreatment for mass spectrometry (MS)-based metabolomics and lipidomics is normally conducted independently with two sample aliquots and separate matrix cleanup procedures, making the two-step process sample-intensive and time-consuming. Herein, we introduce a high-throughput pretreatment workflow for integrated nontargeted metabolomics and lipidomics leveraging the enhanced matrix removal (EMR)-lipid microelution 96-well plates. The EMR-lipid technique was innovatively employed to effectively separate and isolate non-lipid small metabolites and lipids in sequence using significantly reduced sample amounts and organic solvents. Our proposed methodology enables parallel profiling of metabolome and lipidome within a single sample aliquot using ultrahigh-performance liquid chromatography-high resolution mass spectrometry (UHPLC-HRMS). Following method development and optimization with representative metabolites at levels comparable to those detected in human blood, the optimized workflow was applied to prepare metabolome–lipidome from maternal and umbilical cord–blood sera prior to comprehensive profiling using three different UHPLC columns. Results indicate that, compared with conventional two-step metabolomics–lipidomics sample pretreatment workflow, this new approach substantially reduces sample amount and processing time, while still preserving metabolite profiles and revealing additional MS features. Over 2500 metabolites were annotated in human sera with >1000 shared across maternal and cord blood. The shared metabolites are closely linked to various physiological functions, including nutrient transfer, hormonal regulation, waste product clearance, and metabolic programming, underscoring the significant impact of maternal metabolic activities on neonatal metabolic health. In summary, the proposed workflow enables efficient sample pretreatment for nontargeted metabolomics–lipidomics using one single sample while achieving broad metabolite coverage, highlighting its remarkable applicability in clinical and preclinical research.

## Introduction

Metabolomics, the systematic study of metabolites, or low-molecular-weight molecules, endeavors to elucidate the dynamic and intricate metabolic network underlying the key biochemical processes in live biological systems.^1,2^ As a principal field in metabolomics, nontargeted metabolomics can be viewed as a bottom-up holistic strategy that provides comprehensive perspectives on systemic metabolic alterations in response to different stimuli, e.g., disease development, dietary changes, environmental fluctuations, or medical treatments.^2^ Lipidomics, considered as a specialized subdomain of metabolomics, is the comprehensive study of lipidome, encompassing the entire lipid molecules present in cells, organisms, individuals, etc. Metabolomics investigations intuitively aid in understanding the metabolic alterations under physiological and pathological conditions, the discovery of novel therapeutic targets, and the development of personalized medicine.^3^

Although metabolomics technology has been rapidly advancing, various analytical challenges still persist in nontargeted analyses. First, the metabolome encompasses a vast array of metabolites with diverse physicochemical properties and is present at varying concentrations in complex biological matrices. Consequently, challenges arise with respect to ensuring sample quality, minimizing artifacts, standardizing extraction methods, achieving broad metabolome coverage, and accurately annotating metabolites.^4,5^ For lipidomics, in addition to the abovementioned challenges, analysis is further complicated by the difficulty in chromatographic separation and structural elucidation of complex lipid molecules.^6,7^ Additionally, the matrix effect represents another major obstacle to achieving high resolution and broad coverage of metabolites in nontargeted metabolomics. The presence of matrical interferences in biological and environmental samples can lead to ion suppression or enhancement, thereby debilitating analytical reproducibility and reliability.^8^ As such, various strategies are applied to mitigate matrix effects but require substantial additional efforts.^9^ On top of these, the guidelines for nontargeted method validation, data normalization, and integration have not been well established as the validation practices in targeted analysis.^10^

Previously, we and others reported the superior advantages of the enhanced matrix removal (EMR)-lipid sorbent in addressing analytical issues arising from matrix effects.^11,12^ EMR-lipid sorbent stands out among various dispersive SPE (dSPE) sorbents as it selectively traps lipid molecules with long aliphatic chains without retaining the relatively small polar analytes in biosamples.^13−15^ In addition, the 96-well plate format and the microelution pretreatment technique remarkably enhance sample processing efficiency and reproducibility. The EMR-lipid technique has been widely applied in the analysis of non-lipid small metabolites (abbreviated as small metabolites) in multiple types of biosamples, especially lipid-rich materials.^15^ Based on its design features and applications, the EMR-lipid technique can also be utilized for separating lipids and small metabolites in addition to matrix removal. This represents an exciting opportunity for the simultaneous harvesting of small polar metabolites and lipid metabolites with minimal matrix effects prior to metabolomics and lipidomics. In this work, a novel strategy for integrated nontargeted metabolomics and lipidomics was proposed with the application of EMR-lipid as a technique for parallel separation and isolation of the metabolome and lipidome in a single aliquot of biosample. The optimized method was applied to characterize the serum metabolites of healthy pregnant women and the umbilical cord blood of their babies. To the best of our knowledge, this is the first sample pretreatment workflow employing the EMR-lipid technique for integrated nontargeted metabolomics and lipidomics with demonstrated clinical applicability.

## Experimental Section

### Chemicals and Reagents

Description of the solvents, chemicals, and analytical standards used can be found in the Supporting Information.

### Human Blood Collection

Human blood samples were obtained from healthy participants involved in the SmartGen Cohort study consisting of 120 Chinese mother-child pairs. Chinese pregnant women aged 18–45 years without significant medical conditions were recruited during the first trimester of singleton pregnancy during 2018–19.^16^

### Sample Preparation

An aliquot of human serum sample was processed using the proposed EMR-lipid method prior to nontargeted metabolomics and lipidomics analyses, while two aliquots of serum samples were extracted using the conventional methods for comparison.

#### Metabolite Extraction Using EMR-Lipid μelution 96-Well Plates

An aliquot of serum sample (50 μL) was first added to the solvent system MTBE/MeOH/H_2_O (10:3:2.5, v/v/v) for metabolite extraction and deproteinization. In brief, the sample aliquot was sequentially mixed with 300 μL of cold methanol and 1000 μL of cold MTBE and vortexed for 30 s. The mixture was then incubated for 1 h. Next, 250 μL of water was added, and the mixture was incubated for 10 min. After centrifugation, both the upper (MTBE) and lower (MeOH/H_2_O) phases were collected and dried separately using Thermo Scientific Savant SpeedVac concentrator (Waltham, MA, USA). For metabolomics, the dried lower phase was reconstituted in 600 μL of 90% ACN, and the supernatant was transferred to an EMR-lipid 96-well plate and mixed with 100 μL of water containing 4% formic acid. The sorbent was preactivated with 600 μL of 80% ACN containing 4% formic acid. Elution was performed under an increasing vacuum from 1 to 5 in. of mercury (in.Hg), with each step held for 1 min. Following two more rounds of elution with 300 μL of 90% ACN containing 4% formic acid, the eluents were pooled and dried in a SpeedVac concentrator. The dried residue was reconstituted in 150 μL of MeOH/H_2_O (80:20, v/v) and spiked with internal standards (ISs), i.e., 4-chloro-phenylalanine (1 μg/mL) and isotope-labeled standards – Metabolomics QReSS Standard Mix (Cambridge Isotope Laboratories) for metabolomic analysis. To recover lipids trapped in the wells, 620 μL of MTBE/MeOH/H_2_O (10:3:2.5, v/v/v) was added to each well, and elution was conducted under the same vacuum conditions as metabolomics. After two more rounds of elution (310 μL each), the eluents were combined and dried in the SpeedVac concentrator. The dried residue was reconstituted in 150 μL of ACN/IPA/H_2_O (65:30:5, v/v/v) and spiked with CUDA (400 ng/mL) as the IS for lipidomics. The reconstituted extracts were vortexed for 30 s, sonicated for 5 min, and centrifuged at 17,000*g* for 15 min at 4 °C before analysis.

#### Metabolite Extraction Using the Conventional Methods

For metabolomics, 450 μL of ice-cold MeOH was added to an aliquot of serum sample (50 μL) followed by vortexing for 30 s and centrifuged at 17,000*g* for 10 min at 4 °C. For samples with relatively high lipid contents, e.g., blood from obese individuals, samples should proceed to matrix removal before concentrating. The supernatant was dried using a SpeedVac concentrator, spiked with ISs as described above and reconstituted in 150 μL of MeOH/H_2_O (80:20, v/v) and then centrifuged at 17,000*g* for 15 min at 4 °C before analysis.

For lipidomics, lipids were extracted twice using the solvent system MTBE/MeOH/H_2_O (10:3:2.5, v/v/v). In brief, 450 μL of ice-cold methanol was added to a serum aliquot (50 μL) and vortexed followed by the addition of 1000 μL of cold MTBE. After vortexing and incubating for 1 h, 250 μL of water was added to induce phase separation. The sample was centrifuged, and the upper MTBE phase was collected. The lower MeOH phase was re-extracted with 400 μL of the solvent system, and the upper MTBE phase was collected after centrifugation. The combined MTBE phases were dried in a SpeedVac concentrator. The dried lipid residue was spiked with CUDA (400 ng/mL) as IS, reconstituted in 150 μL of ACN/IPA/H_2_O (65:30:5, v/v/v), and then centrifuged before lipidomics analysis.

### UHPLC–HRMS Analysis

The instrumentation consisted of an Orbitrap IQ-X Tribrid mass spectrometer coupled to a Dionex UltiMate 3000 UHPLC system (Thermo Fisher Scientific, Waltham, MA, USA). MS data were acquired in both positive and negative ionization modes. Instrumental calibration was performed using the Pierce FlexMix Calibration Solution (Thermo Fisher Scientific).

For nontargeted metabolomics, chromatographic separation was achieved using an ACQUITY UPLC HSS T3 column (2.1 × 100 mm, 1.8 μm, Waters, Milford, MA, USA). Mobile phase A consisted of 0.1% formic acid in water. Mobile phase B consisted of 0.1% formic acid in ACN. The LC gradient and MS parameters are shown in Table S1. In addition, for very polar small metabolites, an ACQUITY UPLC BEH Amide column (2.1 × 100 mm, 1.7 μm, Waters) was used. Mobile phase A consisted of 5 mM ammonium formate and 0.1% formic acid in water. Mobile phase B consisted of 5 mM ammonium formate, 0.1% formic acid in 95% ACN and 5% water. The LC gradient and MS parameters are shown in Table S2. The column was thermostated at 40 °C during elution. The flow rate was 0.3 mL/min with an injection volume of 3 μL.

For nontargeted lipidomics, chromatographic separation was achieved using an ACQUITY UPLC BEH C18 column (2.1 × 100 mm, 1.7 μm, Waters). Mobile phase A consisted of 5 mM ammonium formate, 0.1% formic acid in 40% ACN and 60% water. Mobile phase B consisted of 5 mM ammonium formate, 0.1% formic acid in 10% ACN and 90% IPA. The column was thermostated at 50 °C during elution. The flow rate was 0.3 mL/min with an injection volume of 3 μL. The LC gradient and MS parameters are shown in Table S3.

### Method Performance Assessment

The following parameters were assessed for different classes of metabolites: recovery, matrix effect, repeatability, and relative abundance. The relative abundance (RA) is calculated as below:
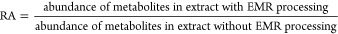


Quality control samples (QCs) were prepared by pooling an small ailquot of each sample (5 μL) for assessing method repeatability and to correct data variations due to signal drifts.^17^ All experiments were conducted in at least triplicate (*n* = 3–6).

### Data Analysis

Mass spectral data for metabolomics were processed using the Compound Discoverer software (v. 3.3, Thermo Fisher Scientific, San Jose, CA, USA) for peak picking, retention time alignment, MS/MS matching, and local/online database searching (e.g., mzCloud and Chemspider) for metabolite annotation. The parameters and settings of the Compound Discoverer software are described in Supporting Information. Lipidomics data was analyzed in Xcalibur QualBrowser (v. 4.5, Thermo Fisher Scientific, San Jose, CA, USA). Subsequently, lipidomics data were processed using LipidSearch software (version 5.0, Thermo Fisher Scientific, San Jose, CA, USA) to identify the lipid molecular species within each lipid fraction. The parameters and settings of the LipidSearch software are described in the Supporting Information. All MS features were filtered based on their coefficient of variation (CV) of integrated peak areas. Only features with a CV below 30% were further examined to avoid highly dispersed data. Lipid metabolites were assigned to major lipid categories according to the LIPID MAPS Lipid Classification System.

Generally, statistical analysis and graph plotting were performed in GraphPad Prism (v10.0, GraphPad Software, Inc., San Diego, CA, USA) and/or RStudio (v. 4.3). A value of *p* < 0.05 was considered statistically significant. Pathway enrichment analysis was performed using MetaboAnalyst 6.0 (https://www.metaboanalyst.ca/ModuleView.xhtml). Log P was predicted by ChemDraw (v20.0, PerkinElmer Informatics, Inc., Waltham, MA, USA). Log P was obtained using ChemSpider (http://www.chemspider.com/) if not available from ChemDraw.

## Results and Discussion

The EMR-lipid technique, featured with a dSPE sorbent, excels at selectively removing various lipid molecules while minimizing the entrapment of small non-lipid analytes from biosamples.^15^ Although the EMR-lipid processing was originally designed for matrix cleanup, we hypothesized that it can be well-suited for metabolite separation and isolation prior to parallel profiling of metabolome (nonlipid) and lipidome, which can be a 2-in-1 integrated process that saves time and sample amount. This study confirmed the feasibility of the EMR-lipid technique for sequentially separating and isolating small metabolites and lipids for integrated parallel nontargeted metabolomics and lipidomics and demonstrated the potential of the proposed workflow in clinical applications.

### Study Design

**Figure 1:**
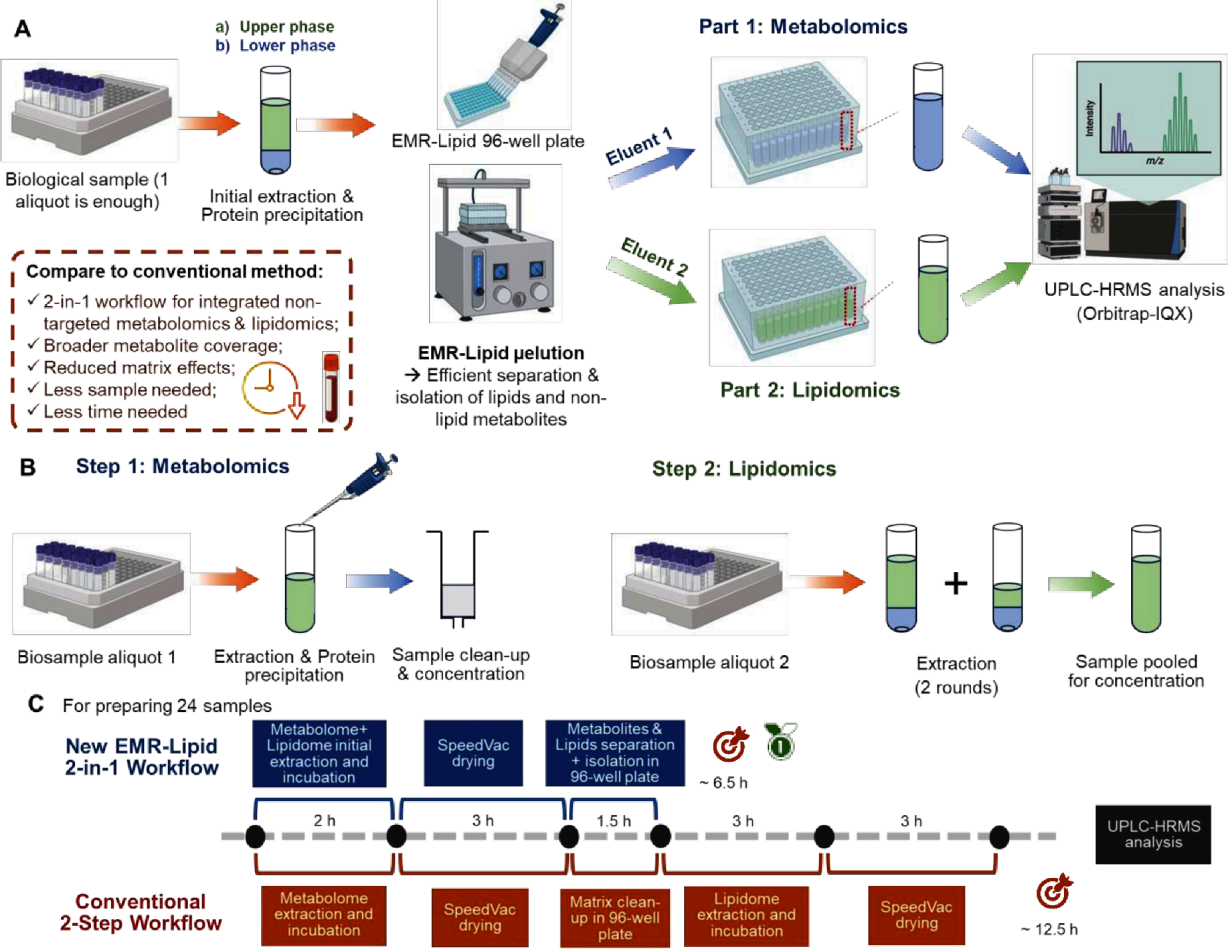
Illustration of the proposed integrated sample pretreatment workflow with EMR-lipid processing (A), and the conventional 2-step workflow for nontargeted metabolomics and lipidomics (B); step-by-step timelines for comparison (C). The initial extraction was performed with MTBE/MeOH/H_2_O (10:3:2.5, v/v/v). “Upper phase” (a) refers to the MTBE layer, while “lower phase” (b) refers to the MeOH/H_2_O layer in text, tables, and figures.

To confirm the feasibility that the small metabolites and lipid metabolites can be separated and eluted in sequence, we first used a biphasic solvent (MTBE + MeOH/H_2_O) to precipitate proteins and to extract as many metabolites as possible and then subject all the extracted metabolites to EMR processing. Through adjusting eluting solvents with different eluotropic strengths and optimizing elution processes, we aimed to retain more lipids in the 96-well plate at the metabolomics step while recovering them mostly at the lipidomics step. Following optimization, method performance was assessed with the use of representative metabolites from the major metabolite superclasses in human blood^18^ (Table S11). Finally, the optimized method was applied to the comparative analysis of human serum metabolome and lipidome from healthy pregnant women and umbilical cord blood of their babies. A comparison of our proposed 2-in-1 workflow versus the traditional two-step metabolomics and lipidomics workflow is illustrated in Figure 1.

### Method Development and Optimization

#### Metabolomics Step

**Figure 2:**
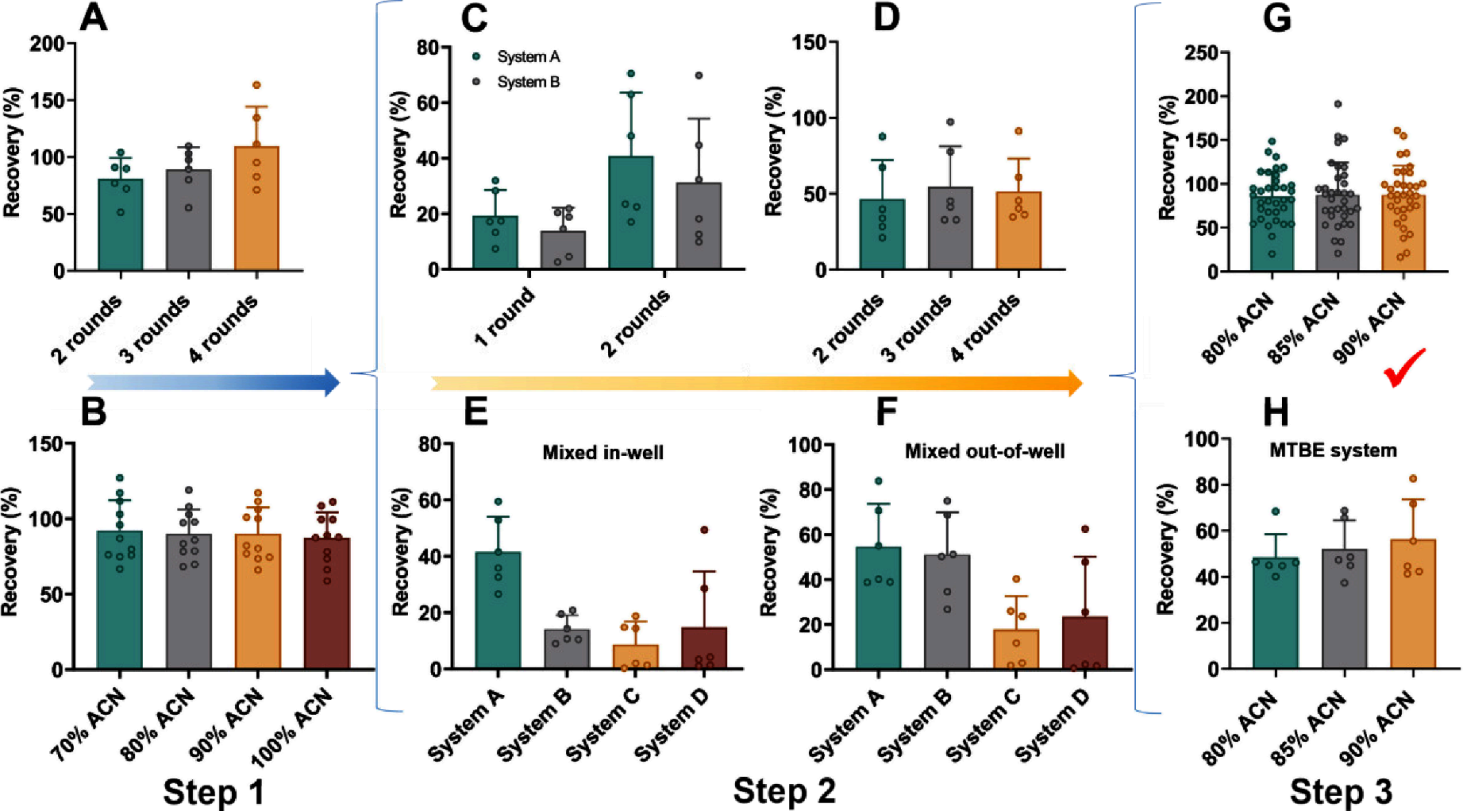
Method development and optimization processes. Step 1: optimization of the metabolomics method: rounds of elution (A) and elution solvents (B). Step 2: optimization of the lipidomics method: rounds of elution (C, D), and elution solvents (E, F). Step 3: optimization of the integrated method for metabolomics and lipidomics: elution solvent systems at the metabolomics step (G), and the lipidomics step (H). Solvent systems: A, MTBE/MeOH/H2O (10:3:2.5, v/v/v); B, CHCl_3_/MeOH/H_2_O (1:2:1, v/v/v); C, CHCl_3_/MeOH/H_2_O (2:1:1, v/v/v); D, CHCl_3_/MeOH/MTBE (3:4:3, v/v/v). Mixed out-of-well, all solvents in the solvent system were mixed outside the EMR-lipid 96-well plate; mixed in-well, solvents were mixed inside the plate.

Building upon our previous work applying the EMR-lipid technique,^15^ the optimization process began with using 80% ACN for eluting non-lipid small metabolites from the loaded EMR 96-well plate. First, we compared the recoveries of representative small metabolites following different rounds of elution (same solvent volume for each round). As shown in Figure 2A, 3 rounds of elution obtained better average recovery (closer to 100%) for most metabolites tested than 2 or 4 rounds of elution. Although the four-round elution showed elevated recoveries, additional elution increased the chances of eluting lipid metabolites at the metabolomics step and also made the entire process more time-consuming. Therefore, a three-round elution was selected for further optimization. As shown in Table S4, satisfactory recoveries (80–100%) were achieved for most representative metabolites of diverse classes at this step, particularly promising for amino acids (86%), nucleotides, purine and derivatives (80%), energy metabolism intermediates (103%), xenobiotics (109%), and bile acids (100%). Next, we compared the effects of elution solvents with different percentages of ACN in water on the recoveries of metabolites following three rounds of elution. As shown in Figure 2B and Table S5, the recoveries of metabolites eluted with 70–100% ACN were largely comparable. Yet, for certain metabolites, e.g., l-leucine, l-valine, l-carnitine, adenine, and cholic acid, elution with 80% and 90% ACN allowed better recoveries than 70% ACN. Therefore, 80% and 90% ACN were selected as the preferred solvents for further assessment at the method integration step.

#### Lipidomics Step

According to our preliminary study, the primary challenge lay in the recovery of the adsorbed lipids in the 96-well plate. For lipid extraction, the most commonly used solvent systems include CHCl_3_-based (e.g., Folch, Bligh, and Dyer) and MTBE-based systems.^19^ In order to confirm the feasibility of recovering lipids trapped following the previous metabolomics elution step, we mixed a stock solution of 6 representative lipids with other small metabolites, diluted it to levels comparable to endogenous concentrations, and loaded it onto the EMR plate. First, we compared the lipid-eluting power of classical MTBE-based System A (MTBE/MeOH/H_2_O, 10:3:2.5, v/v/v) and CHCl_3_-based System B (CHCl_3_/MeOH/H_2_O, 2:1:1, v/v/v). As shown in Figure 2C and Table S6, System A gave better recoveries than System B, and two rounds resulted in better recoveries than one round of elution. Furthermore, we compared the recoveries with 2–4 rounds of elution using MTBE-based System A. As shown in Figure 2D and Table S7, three rounds of elution gave the best recoveries. For example, three-round elution (97.2%) showed the best recovery for 15:0–18:1 PE when compared with two-round elution (87.6%) and four-round elution (91.2%). More recently, a one-phase extraction system consisting of CHCl_3_, MeOH, and MTBE (System D) was introduced to extract lipids for lipidomics.^19^ Therefore, we further compared the efficiency of four solvent systems in releasing trapped lipids from the wells. As shown in Figure 2E,F and Tables S8, the best recoveries for most lipids tested were achieved with System A and mixing out-of-well. By contrast, mixing the solvents in the system in the well resulted in considerably lower recoveries.

#### Method Integration

Following optimization of each step, the workflow processes were integrated for both metabolomics and lipidomics. Additional metabolite standards were included to mimic the representative metabolite classes and concentration levels present in human blood. To further optimize the integrated method, three elution solvents for small metabolites, i.e., 80%, 85%, and 90% ACN, were further compared at the metabolomics step. As shown in Figure 2G and Table S9, 85% and 90% ACN resulted in better recoveries for most metabolites tested compared to 80% ACN. In addition, the highest recoveries of representative lipids at the lipidomics step were achieved when 90% ACN was used at the metabolomics step (Figure 2H and Table S10). Results suggest that 90% ACN was the optimal elution solvent at the metabolomics step, although the recoveries of different metabolites, even within the same class, still varied a lot. For example, among hydrophobic amino acids, l-leucine and l-valine had good recoveries, while l-alanine and l-proline were much less satisfactory. A similar phenomenon was also observed in other classes such as acylcarnitines, nucleotides, purines and derivatives, and xenobiotics. For lipidomics, 18:1 Lyso PE and 15:0–18:1 PE were better recovered than the other lipid groups. This led us to further examine the performance of this workflow, with respect to the recoveries of different classes of metabolites in both solvent systems and human serum samples.

### Method Performance Assessment

Analytical method performance is crucial for metabolomics to ensure high data quality for subsequent reliable biological interpretation and translational application of the findings. There are very limited references to nontargeted metabolomics method performance. Similar to targeted metabolomics, method performance should be assessed prior to application of nontargeted analysis.^17^ In this study, method performance was assessed in terms of recovery, matrix effect, repeatability, and relative abundance (RA).

#### Assessment of Recovery, Matrix Effect, and Repeatability

Using the optimized workflow, we determined the recoveries of representative human blood metabolites first in pure solvent systems. Results show that more than half of the tested metabolites (20 of 32) exhibited satisfactory recoveries (80–120%) (Table S11). Furthermore, to determine the recovery of metabolites in the presence of biomatrix, 12 isotope-labeled ISs representing the major metabolite superclasses in human sera with diverse chemical properties and molecular masses were spiked before (prespiked) or after (postspiked) the entire pretreatment process. The recovery was assessed by comparing the peak area of isotope-labeled ISs in the prespiked samples to that in the postspiked samples. As shown in Table S12, the method resulted in good recoveries for almost all of the ISs. Meanwhile, the matrix effect was assessed by comparing the recoveries of ISs spiked in seral extracts following EMR-lipid processing with that processed without EMR-lipid. Almost all ISs experienced insignificant matrix effect. The repeatability, or intraday precision, was determined as the RSD (%, *n* = 6). According to the FDA guideline, the threshold of repeatability was set at 15%.^20^ All analytes exhibited satisfactory repeatability with RSDs < 15% (Table S12**)**.

#### Influence of EMR-Lipid Processing on the Recovery and Coverage of Metabolites in Human Serum

**Figure 3:**
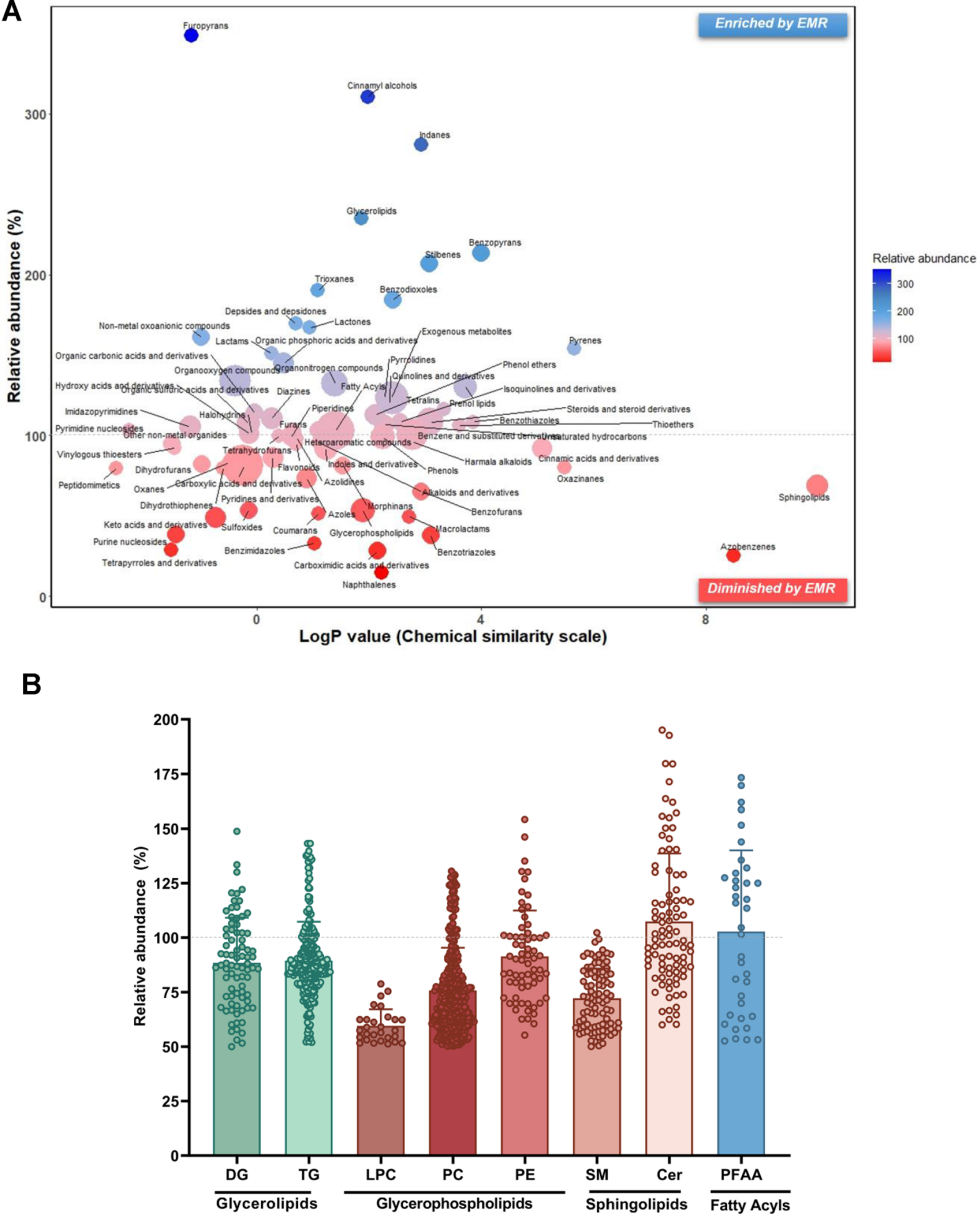
Influence of EMR-lipid processing on the recovery (stated as relative abundance) of different classes of metabolites in human sera. Relative abundance (%) of annotated metabolites eluted at the metabolomics step (A) or the lipidomics step (B) with EMR-lipid processing compared to that without the processing. 516 annotated small metabolites were clustered according to their chemical taxonomy recorded in the Human Metabolome database (HMDB). *X*-axis is the mean logarithmic octanol–water partition coefficients (log P) with the node size indicating total compound numbers for each cluster set and node color depicting the varying degrees of relative abundance (blue–enriched, red–diminished); *Y*-axis is the mean relative abundance for each cluster set. Refer to Table S18 for full names of lipid subclasses.

Next, we assessed the overall influence of EMR-lipid processing on the recovery of different classes of metabolites in human sera from healthy Chinese pregnant women, which is expressed as relative abundance (RA), the ratio of metabolite abundance/peak intensity in samples with or without EMR processing (Figure 3, Tables S13 and S14). At the metabolomics step, 606 metabolites with relatively high abundances (peak area >10^6^) were annotated with 516 metabolites exhibiting relatively good RA (RSD < 30%, Figure 3A). 270 metabolites with satisfactory RA (75–125%) are listed in Table S13. These metabolites mainly belong to carboxylic acids and derivatives (84 annotated), organooxygen compounds (40), benzene and substituted derivatives (38), steroids and steroid derivatives (25), organonitrogen compounds (21), phenols (14), etc. Regarding the annotated lipid metabolites, 1137 metabolites with RA values ranging 50–200% and RSD < 30% were selected for further investigation (Figure 3B). Consistent with previous reports, the lipids identified in our study mainly belong to six lipid classes, i.e., glycerolipids, glycerophospholipids, sphingolipids, prenols, sterols, and fatty acyls.^21^ Among them, 592 lipids achieved satisfactory RA (75–125%), primarily from the classes of diradylglycerol (DG), triradylglycerol (TG), phosphatidylcholine (PC), ceramide (Cer), phosphatidylethanolamine (PE), and sphingomyelin (SM) (Table S14). Of note, some categories of lipids were also detected at considerable amounts at the metabolomics step, including 25 sterols, 10 prenol lipids, and 74 fatty acyls (Figure 3A).

**Figure 4:**
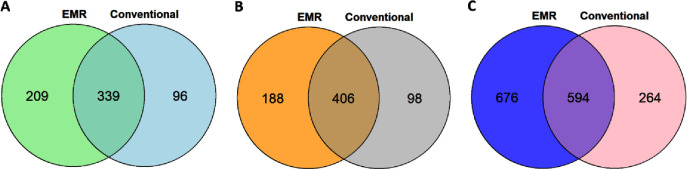
Comparison of metabolite coverage in human serum using the proposed and conventional methods. Metabolite extracts prepared using the EMR-lipid workflow (left circle) or the conventional workflow (right circle) were chromatographically separated by three UHPLC columns: BEH Amide (A) and HSS T3 (B) for metabolomics; BEH C18 (C) for lipidomics.

In terms of metabolite coverage, using the new workflow, a broader range of metabolites were revealed, with 615 additional metabolites uncovered compared with the conventional workflow (Figure 4). All these demonstrate that the integrated EMR-lipid workflow can effectively separate small metabolites from lipids, which enables sequential isolation and comprehensive profiling of metabolites within a single sample aliquot, achieving satisfactory recoveries, insignificant matrix effect, and broad coverage. This will enable comprehensive metabolomic fingerprinting with high consistency, allowing for a more in-depth exploration of the alterations in metabolic network and the identification of novel biomarkers.

#### Assessment of the Lipids Recovered in the Lower Phase by the EMR Processing

**Figure 5:**
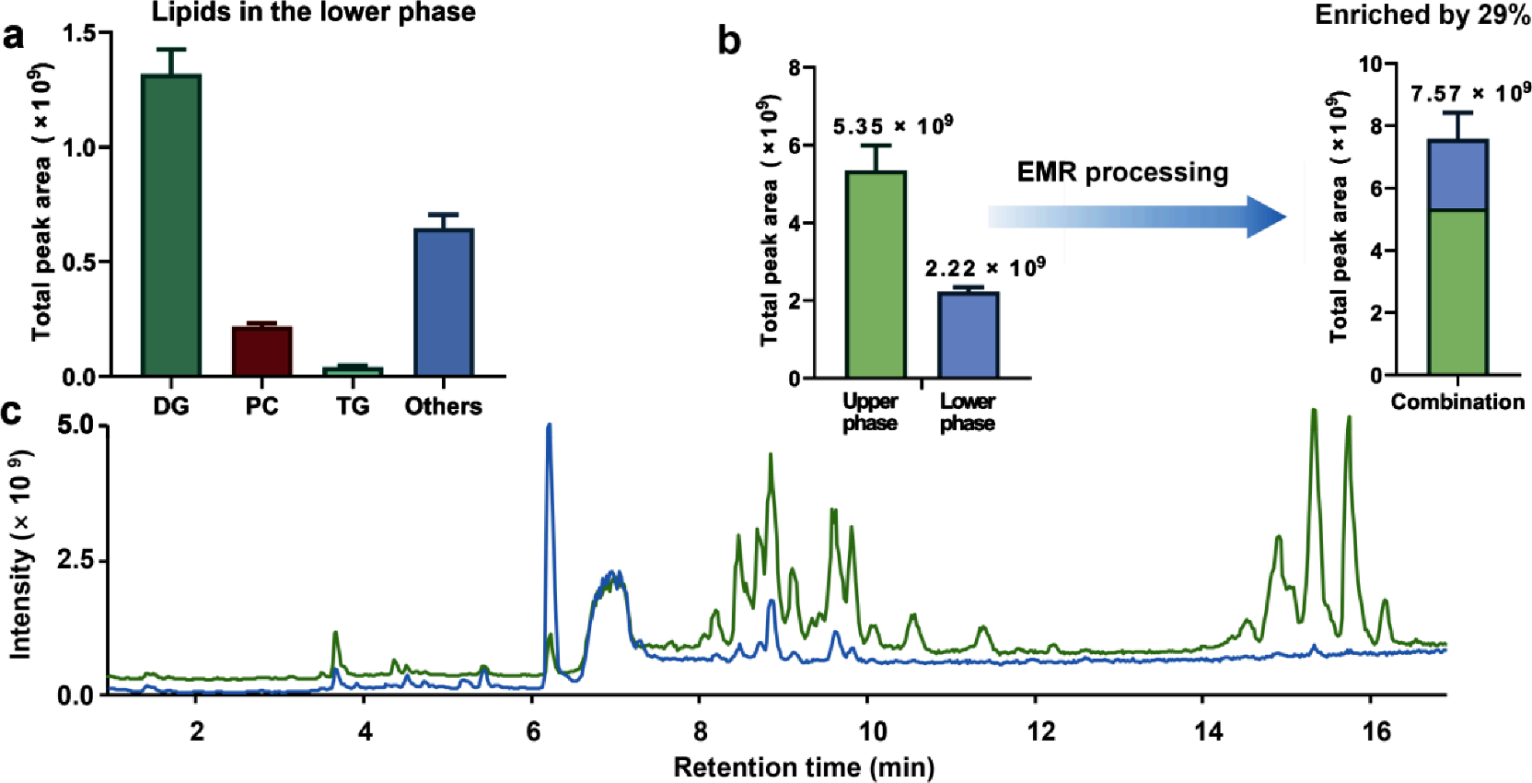
Analysis of lipids from the upper and lower phases indicates the effects of EMR-lipid processing. (a) Abundance (expressed as total peak intensity) of different classes of lipids present in the lower phase (recovered following EMR processing). Abundance (b) and total ion chromatogram (c) of the lipids recovered in the lower phase (MeOH/H_2_O layer–blue trace) compared with the lipids in the upper phase (MTBE layer–green trace). Refer to Table S18 for full names of lipid classes.

Compared with the pretreatment method with EMR-lipid processing, the conventional 2-in-1 pretreatment method for metabolomics and lipidomics has some drawbacks. It should be noted that the upper phase, mainly MTBE, is truth mixed with a small amount of methanol and possibly even water, while the lower phase, mainly methanol, may also contain MTBE. Some lipids in the lower phase (MeOH/H_2_O layer) can be lost if only the upper phase (MTBE layer) was analyzed at the lipidomics step. Meanwhile, the lipids present will cause matrix effects when the lower phase was subjected to HRMS analysis. Therefore, we determined the lipids eluted at the metabolomics step and the small metabolites lost at the lipidomics steps to gain a more comprehensive view on method performance. As shown in Figure 5 and Table S15, different classes of lipids were detected in the lower phase. Considering that a significant amount of lipids (ca. 29%) was lost to the lower phase, it is necessary to combine the lipids recovered from the lower phase (via EMR processing) with those extracted into the upper phase for lipidomics. Additionally, we also detected a number of small metabolites present in the upper phase (Table S16). However, as they represent less than 6% of total small metabolites, the workflow was simplified to only subject the lower phase to EMR-lipid processing for recovering more lipids which are then combined with MTBE-extracted lipids (Figure 1).

### Method Application – Comparative Analysis of Maternal and Umbilical Cord Blood Metabolomes

**Figure 6:**
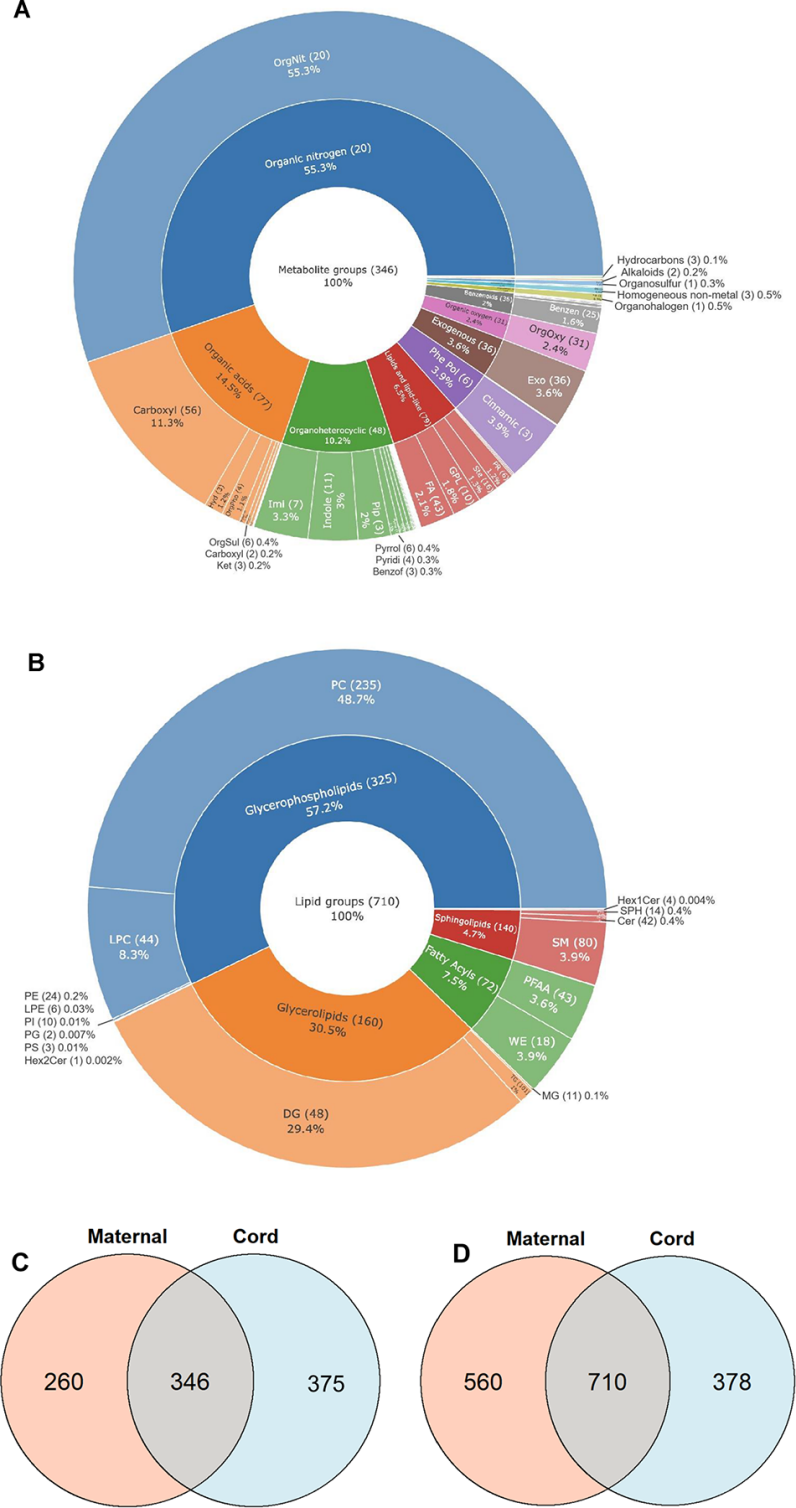
Classification and distribution of shared metabolites across maternal and cord blood sera. (A) Shared metabolites eluted at the metabolomics step (346 in total), primarily categorized into 11 major metabolite classes and 57 subclasses (refer to Table S17 for the full names). (B) Shared lipids eluted at the lipidomics step (710 in total), primarily categorized into 4 major lipid classes and 19 subclasses (refer to Table S18 for full names). Venn diagrams show the number of distinctive and shared small metabolites (C) and lipid metabolites (D).

The developed method was further applied to comprehensive profiling of the metabolome and lipidome in human serum samples from healthy Chinese puerperants and their corresponding cord blood. As the human blood metabolome encompasses metabolites of vastly diverse polarity, BEH amide (for very polar compounds), HSS T3 (for polar and moderately polar compounds), and BEH C18 (for nonpolar and moderately polar compounds) UHPLC columns were used in combination. We annotated a total of 2629 small metabolites and lipids, of which 1056 were shared across maternal and cord blood. For small metabolites, 606 compounds were identified in maternal blood and 721 in cord blood, with 346 shared metabolites categorized into 57 classes, represented by carboxylic acids and derivatives, fatty acyls, and organooxygen compounds (Figure 6A). In addition, global lipidome profiling revealed 1270 lipids in maternal sera and 1088 lipids in cord blood sera (Figure 6B). Among these, 710 lipids were identified as shared metabolites, consisting of 235 PCs, 101 TGs, 80 SMss, 48 DG, and 44 LPCs. Previous findings on maternal and cord blood metabolomic profiles are summarized in Table 1. Compared with all previous studies, the metabolite coverage in this study was significantly expanded with the application of the proposed EMR-lipid workflow. This highlights the significant potential of our sample pretreatment method in identifying novel biomarkers for evaluating maternal-fetal health and predicting disease risks. Although our cohort samples were from healthy individuals, the shared metabolites still reflect the metabolite exchange between the puerperants and umbilical cord. The shared small metabolites can be classified according to their physiological functions related to maternal and child health: nutrient transfer (e.g., choline, dl-tryptophan, niacin, and taurochenodeoxycholic acid), hormonal regulation (e.g., progesterone and cortisol), waste product removal (e.g., creatinine and uric acid), and metabolic programming (e.g., 5-hydroxyindoleacetic acid and spermine).^32−36^ In the context of metabolic programming, work by the Shokry workflow demonstrated a positive association between body mass index (BMI) and levels of leucine and isoleucine, while gestational diabetes mellitus (GDM) was linked to the elevated hexoses levels in both maternal and cord blood.^23^ Moros et al. found that elevated levels of branched chain amino acids (leucine, isoleucine and valine) in intrauterine growth-restricted pregnancies correlated with increased insulin resistance based on maternal and umbilical cord blood metabolomics analysis.^22^ In the lipidome, PC and DG were identified as the most abundant shared lipid classes. PCs are essential for fetal development, particularly in the growth and maturation of the fetal brain and nervous system, while DGs play significant roles in insulin sensitivity, which is vital for glucose regulation and maintaining the metabolic health of both the mother and fetus.^37^ Furthermore, other abundant shared metabolites such as androsterone sulfate and 16α-hydroxydehydroepiandrosterone 3-sulfate have been infrequently studied in the context of maternal and child health yet may possess significant implications. Pathway enrichment analysis also highlighted the involvement of these shared metabolites in multiple metabolic pathways, e.g., alanine, aspartate, and glutamate metabolism, steroid hormone biosynthesis, primary bile acid biosynthesis and sphingolipid metabolism (Figure S1).

**Table 1:** Summary of the Numbers of Maternal and Cord Blood Metabolites Annotated in the Present and Previous Relevant Studies

sample type	metabolites annotated	shared metabolites	refs
maternal and cord (serum)	2629	1056	present study
metabolomics analysis^a^			
maternal and cord (serum)	181	56	Moros et al. 2021^22^
maternal and cord (plasma)	400	201	Shokry et al. 2019^23^
maternal and cord (serum)	n.a.^b^	37	Shearer et al. 2021^24^
maternal and cord (serum)	n.a.^b^	203	Zhu et al. 2023^25^
cord (serum)	68		Robinson et al. 2018^26^
cord (plasma)	230		Schlueter et al. 2020^27^
cord (plasma)	155		Ross et al. 2021^28^
cord (plasma)	125		Hartvigsson et al. 2022^29^
lipidomics analysis			
maternal and cord (plasma)	n.a.^b^	573	LaBarre et al. 2020^30^
maternal and cord (plasma)	480	480	LaBarre et al. 2020^31^

a Metabolome and lipidome were not separately extracted and were profiled in one sample extract.

b n.a., data is not available.

While our results highlight the distinct advantages of the integrated EMR-lipid workflow, some limitations should be acknowledged. For example, analytical reproducibility in the workflow has not been thoroughly investigated, especially when using different MS instrumentation platforms (e.g., AB Sciex QTRAP 5500 MS vs Thermo Fisher Scientific Q Exactive HRMS) in different laboratories or research centers. In addition, issues with low relative abundances of certain metabolite and lipid classes warrant further method optimization or can be applied in conjunction with targeted analysis. Future cross-laboratory validation shall further demonstrate the feasibility of this novel approach in broad preclinical and clinical applications.

## Conclusions

This study presents a novel workflow utilizing the EMR-lipid technique for sequential separation and isolation of non-lipid small metabolites and lipid metabolites prior to integrated nontargeted metabolomics and lipidomics. Compared with the conventional 2-step metabolomic and lipidomic profiling, parallel profiling of small metabolites and lipids in a single aliquot of biosample captures more comprehensive and reliable information on the metabolic landscape. This novel method also reduces the amount of biosamples needed (up to 50%) while achieving efficient and wide-coverage analysis (increasing the overall metabolite coverage by over 34%). The EMR-lipid workflow also holds significant promise for automation, which can be easily integrated into efficient and robust clinical and preclinical metabolomics and lipidomics pipelines. In future investigations, application of this novel analytical workflow to larger human cohorts is anticipated to substantially deepen our understanding on how changes in metabolite profile or certain metabolite(s) reflect or influence the multifaceted spectra of human health.

## Supporting Information Available

Chemicals and reagents; instrumentation and LC-MS parameters; Table S1: UHPLC-HRMS parameters using the HSS T3 column at the metabolomics step; Table S2: UHPLC-HRMS parameters using the BEH amide column at the metabolomicsstep; Table S3: UHPLC-HRMS parameters using the BEH C18 column at the lipidomics step; data analysis; Table S4: the recoveries of non-lipid metabolites following two, three and four rounds of elution; Table S5: the recoveries of non-lipid metabolites following different elution solvents; Table S6: the recoveries of lipids using two solvent systems with one and two rounds of elution; Table S7: the recoveries of lipids using two, three or four rounds of elution at the lipidomics step; Table S8: the recoveries of lipids eluted using different solvent systems and mixing modes; Table S9: absolute recoveries of non-lipid metabolites at the metabolomics step using different elution solvents; Table S10: absolute recoveries of representative lipid metabolites with the different solvents for eluting metabolites at the metabolomics step; Table S11: recoveries of representative metabolites present in human blood using the optimized method; Table S12: recoveries, matrix effect, and repeatability assessed using isotope-labeled internal standards; Table S13: the recovery (expressed as relative abundance) of annotated metabolites eluted at the metabolomics step with EMR-lipid processing compared to that without processing; Table S14: the relative recovery (expressed as relative abundance) of annotated lipids at the lipidomics step with EMR-lipid processing compared to that without the processing; Table S15: the high-abundance lipids recovered in the lower phase following EMR-lipid processing; Table S16: the small metabolites detected in the upper phase of MTBE extracts; Table S17: list of full names for non-lipid small metabolite classes reported in Figure 6A; Table S18: list of full names for lipid metabolite classes reported in Figure 6B; Figure S1: pathway enrichment analysis of the annotated metabolites shared across maternal and cord blood sera (PDF)

## References

[ref1] Patti G. J.; Yanes O.; Siuzdak G. Metabolomics: the apogee of the omics trilogy. Nat. Rev. Mol. Cell Biol. 2012, 13 (4), 263–269. 10.1038/nrm3314. 22436749 PMC3682684

[ref2] Qiu S.; Cai Y.; Yao H.; Lin C.; Xie Y.; Tang S.; Zhang A. Small molecule metabolites: discovery of biomarkers and therapeutic targets. Signal Transduction Targeted Ther. 2023, 8 (1), 132 10.1038/s41392-023-01399-3. PMC10026263 36941259

[ref3] Barri T.; Holmer-Jensen J.; Hermansen K.; Dragsted L. O. Metabolic fingerprinting of high-fat plasma samples processed by centrifugation-and filtration-based protein precipitation delineates significant differences in metabolite information coverage. Anal. Chim. Acta 2012, 718, 47–57. 10.1016/j.aca.2011.12.065. 22305897

[ref4] Chaleckis R.; Meister I.; Zhang P.; Wheelock C. E. Challenges, progress and promises of metabolite annotation for LC–MS-based metabolomics. Curr. Opin. Biotechnol. 2019, 55, 44–50. 10.1016/j.copbio.2018.07.010. 30138778

[ref5] Dudzik D.; Barbas-Bernardos C.; García A.; Barbas C. Quality assurance procedures for mass spectrometry untargeted metabolomics. a review. J. Pharm. Biomed. Anal. 2018, 147, 149–173. 10.1016/j.jpba.2017.07.044. 28823764

[ref6] Li J.; Vosegaard T.; Guo Z. Applications of nuclear magnetic resonance in lipid analyses: An emerging powerful tool for lipidomics studies. Prog. Lipid Res. 2017, 68, 37–56. 10.1016/j.plipres.2017.09.003. 28911967

[ref7] Bonney J. R.; Prentice B. M. Perspective on emerging mass spectrometry technologies for comprehensive lipid structural elucidation. Anal. Chem. 2021, 93 (16), 6311–6322. 10.1021/acs.analchem.1c00061. 33856206 PMC8177724

[ref8] Trufelli H.; Palma P.; Famiglini G.; Cappiello A. An overview of matrix effects in liquid chromatography–mass spectrometry. Mass Spectrom. Rev. 2011, 30 (3), 491–509. 10.1002/mas.20298. 21500246

[ref9] Cortese M.; Gigliobianco M. R.; Magnoni F.; Censi R.; Di Martino P. Compensate for or minimize matrix effects? Strategies for overcoming matrix effects in liquid chromatography-mass spectrometry technique: a tutorial review. Molecules 2020, 25 (13), 3047 10.3390/molecules25133047. 32635301 PMC7412464

[ref10] Misra B. B. Data normalization strategies in metabolomics: Current challenges, approaches, and tools. Eur. J. Mass Spectrom. 2020, 26 (3), 165–174. 10.1177/1469066720918446. 32276547

[ref11] Yang Y.; Lee P.-K.; Wong H.-C.; Zhao D. Oral supplementation of Gordonibacter urolithinfaciens promotes ellagic acid metabolism and urolithin bioavailability in mice. Food Chem. 2024, 437, 137953 10.1016/j.foodchem.2023.137953. 37976786

[ref12] Zhao D.; Yuan B.; Kshatriya D.; Polyak A.; Simon J. E.; Bello N. T.; Wu Q. Influence of diet-induced obesity on the bioavailability and metabolism of raspberry ketone (4-(4-Hydroxyphenyl)-2-Butanone) in mice. Mol. Nutr. Food Res. 2020, 64 (8), 1900907 10.1002/mnfr.201900907. PMC7329366 32052560

[ref13] Hakme E.; Lozano A.; Uclés S.; Gómez-Ramos M. M.; Fernández-Alba A. R. High-throughput gas chromatography-mass spectrometry analysis of pesticide residues in spices by using the enhanced matrix removal-lipid and the sample dilution approach. J. Chromatogr. A 2018, 1573, 28–41. 10.1016/j.chroma.2018.08.046. 30224277

[ref14] López-Blanco R.; Nortes-Méndez R.; Robles-Molina J.; Moreno-González D.; Gilbert-López B.; García-Reyes J. F.; Molina-Díaz A. Evaluation of different cleanup sorbents for multiresidue pesticide analysis in fatty vegetable matrices by liquid chromatography tandem mass spectrometry. J. Chromatogr. A 2016, 1456, 89–104. 10.1016/j.chroma.2016.06.019. 27328883

[ref15] Yuan B.; Zhao D.; Lyu W.; Yin Z.; Kshatriya D.; Simon J. E.; Bello N. T.; Wu Q. Development and validation of a micro-QuEChERS method with high-throughput enhanced matrix removal followed with UHPLC-QqQ-MS/MS for analysis of raspberry ketone-related phenolic compounds in adipose tissues. Talanta 2021, 235, 122716 10.1016/j.talanta.2021.122716. 34517584 PMC8441007

[ref16] Chen Y.; Chiou A. J.; Leung A. S. Y.; Chan K. C. C.; Chang M. K.; Cheng N. S.; Chan P. K. S.; Wong M. S.; Tam W. H.; Leung T. F. Human milk oligosaccharides in Chinese lactating mothers and relationship with allergy development in offspring. Asian Pac. J. Allergy Immunol. 2023. 10.12932/AP-110922-1453 37061931

[ref17] Kouassi Nzoughet J.; Bocca C.; Simard G.; Prunier-Mirebeau D.; Chao de la Barca J. M.; Bonneau D.; Procaccio V.; Prunier F.; Lenaers G.; Reynier P. A nontargeted UHPLC-HRMS metabolomics pipeline for metabolite identification: application to cardiac remote ischemic preconditioning. Anal. Chem. 2017, 89 (3), 2138–2146. 10.1021/acs.analchem.6b04912. 27992159

[ref18] Lawton K. A.; Berger A.; Mitchell M.; Milgram K. E.; Evans A. M.; Guo L.; Hanson R. W.; Kalhan S. C.; Ryals J. A.; Milburn M. V. Analysis of the Adult Human Plasma Metabolome. Pharmacogenomics 2008, 9 (4), 383–397. 10.2217/14622416.9.4.383. 18384253

[ref19] Gil A.; Zhang W.; Wolters J. C.; Permentier H.; Boer T.; Horvatovich P.; Heiner-Fokkema M. R.; Reijngoud D.-J.; Bischoff R. One-vs two-phase extraction: re-evaluation of sample preparation procedures for untargeted lipidomics in plasma samples. Anal. Bioanal. Chem. 2018, 410, 5859–5870. 10.1007/s00216-018-1200-x. 29968103 PMC6096717

[ref20] FDA, U. S. Q2(R2) Validation of Analytical Procedures; 2024. https://www.fda.gov/regulatory-information/search-fda-guidance-documents/q2r2-validation-analytical-procedures (accessed March, 2024).

[ref21] Tabassum R.; Ripatti S. Integrating lipidomics and genomics: emerging tools to understand cardiovascular diseases. Cell. Mol. Life Sci. 2021, 78, 2565–2584. 10.1007/s00018-020-03715-4. 33449144 PMC8004487

[ref22] Moros G.; Boutsikou T.; Fotakis C.; Iliodromiti Z.; Sokou R.; Katsila T.; Xanthos T.; Iacovidou N.; Zoumpoulakis P. Insights into intrauterine growth restriction based on maternal and umbilical cord blood metabolomics. Sci. Rep. 2021, 11 (1), 7824 10.1038/s41598-021-87323-7. 33837233 PMC8035183

[ref23] Shokry E.; Marchioro L.; Uhl O.; Bermúdez M. G.; García-Santos J. A.; Segura M. T.; Campoy C.; Koletzko B. Impact of maternal BMI and gestational diabetes mellitus on maternal and cord blood metabolome: results from the PREOBE cohort study. Acta Diabetol. 2019, 56, 421–430. 10.1007/s00592-019-01291-z. 30725264

[ref24] Shearer J.; Klein M. S.; Vogel H. J.; Mohammad S.; Bainbridge S.; Adamo K. B. Maternal and cord blood metabolite associations with gestational weight gain and pregnancy health outcomes. J. Proteome Res. 2021, 20 (3), 1630–1638. 10.1021/acs.jproteome.0c00854. 33529033

[ref25] Zhu M.; Sun R.; Jin L.; Yu D.; Huang X.; Zhu T.; Gong Y.; Chen Y.; Shi J.; Wang Q.; Lu C.; Wang D. Metabolomics profiling of maternal and umbilical cord blood in normoglycemia macrosomia. J. Matern.-Fetal Neonat. Med. 2023, 36 (2), 2270761 10.1080/14767058.2023.2270761. 37848386

[ref26] Robinson O.; Keski-Rahkonen P.; Chatzi L.; Kogevinas M.; Nawrot T.; Pizzi C.; Plusquin M.; Richiardi L.; Robinot N.; Sunyer J.; Vermeulen R.; Vrijheid M.; Vineis P.; Scalbert A.; Chadeau-Hyam M. Cord blood metabolic signatures of birth weight: a population-based study. J. Proteome Res. 2018, 17 (3), 1235–1247. 10.1021/acs.jproteome.7b00846. 29401400

[ref27] Schlueter R. J.; Al-Akwaa F. M.; Benny P. A.; Gurary A.; Xie G.; Jia W.; Chun S. J.; Chern I.; Garmire L. X. Prepregnant obesity of mothers in a multiethnic cohort is associated with cord blood metabolomic changes in offspring. J. Proteome Res. 2020, 19 (4), 1361–1374. 10.1021/acs.jproteome.9b00319. 31975597 PMC7357408

[ref28] Ross A. B.; Barman M.; Hartvigsson O.; Lundell A.-C.; Savolainen O.; Hesselmar B.; Wold A. E.; Sandberg A.-S. Umbilical cord blood metabolome differs in relation to delivery mode, birth order and sex, maternal diet and possibly future allergy development in rural children. PLoS One 2021, 16 (1), e0242978 10.1371/journal.pone.0242978. 33493154 PMC7833224

[ref29] Hartvigsson O.; Barman M.; Savolainen O.; Ross A. B.; Sandin A.; Jacobsson B.; Wold A. E.; Sandberg A.-S.; Brunius C. Differences between Arterial and Venous Umbilical Cord Plasma Metabolome and Association with Parity. Metabolites 2022, 12 (2), 175 10.3390/metabo12020175. 35208249 PMC8877791

[ref30] LaBarre J. L.; Puttabyatappa M.; Song P. X. K.; Goodrich J. M.; Zhou L.; Rajendiran T. M.; Soni T.; Domino S. E.; Treadwell M. C.; Dolinoy D. C.; Padmanabhan V.; Burant C. F. Maternal lipid levels across pregnancy impact the umbilical cord blood lipidome and infant birth weight. Sci. Rep. 2020, 10 (1), 14209 10.1038/s41598-020-71081-z. 32848180 PMC7449968

[ref31] Mir S. A.; Chen L.; Burugupalli S.; Burla B.; Ji S.; Smith A. A. T.; Narasimhan K.; Ramasamy A.; Tan K. M.-L.; Huynh K.; et al. Population-based plasma lipidomics reveals developmental changes in metabolism and signatures of obesity risk: a mother-offspring cohort study. BMC Med. 2022, 20 (1), 242 10.1186/s12916-022-02432-y. 35871677 PMC9310480

[ref32] Badawy A. A.-B. Tryptophan metabolism, disposition and utilization in pregnancy. Biosci. Rep. 2015, 35 (5), e00261 10.1042/BSR20150197. 26381576 PMC4626867

[ref33] Rinne G. R.; Hartstein J.; Guardino C. M.; Schetter C. D. Stress before conception and during pregnancy and maternal cortisol during pregnancy: A scoping review. Psychoneuroendocrinology 2023, 153, 106115 10.1016/j.psyneuen.2023.106115. 37119659 PMC10936734

[ref34] Dickinson H.; Bain E.; Wilkinson D.; Middleton P.; Crowther C. A.; Walker D. W. W. Creatine for women in pregnancy for neuroprotection of the fetus. Cochrane Database Syst. Rev. 2014, CD010846 10.1002/14651858.CD010846.pub2. 25523279 PMC10657457

[ref35] Zhou G.; Holzman C.; Luo Z.; Margerison C. Maternal serum uric acid levels in pregnancy and fetal growth. Journal of Maternal-Fetal & Neonatal Medicine 2020, 33 (1), 24–32. 10.1080/14767058.2018.1484093. 29961396

[ref36] Staud F.; Pan X.; Karahoda R.; Dong X.; Kastner P.; Horackova H.; Vachalova V.; Markert U. R.; Abad C. Characterization of a human placental clearance system to regulate serotonin levels in the fetoplacental unit. Reprod. Biol. Endocrinol. 2023, 21 (1), 74 10.1186/s12958-023-01128-z. 37612712 PMC10464227

[ref37] Derbyshire E.; Obeid R. Choline, neurological development and brain function: a systematic review focusing on the first 1000 days. Nutrients 2020, 12 (6), 1731 10.3390/nu12061731. 32531929 PMC7352907

