# Supplementary material for: A High-Throughput Integrated Nontargeted Metabolomics and Lipidomics Workflow Using Microelution Enhanced Matrix Removal-Lipid for Comparative Analysis of Human Maternal and Umbilical Cord Blood Metabolomes
Source: Anal Chem. 2025 Jan 30;97(5):2629–38. doi: 10.1021/acs.analchem.4c03222 (PMC11822729; doi:10.1021/acs.analchem.4c03222)
Supplement: Supplementary file 1 — ac4c03222_si_001.pdf [file ac4c03222_si_001.pdf]

Supporting Information

A High-Throughput Integrated Nontargeted Metabolomics and Lipidomics Workflow Using Microelution Enhanced Matrix Removal-Lipid for Comparative Analysis of Human Maternal and Umbilical Cord Blood Metabolomes

Wenjie Wu [a,b], Ke Wang [a,b,f], Jianing Liu [a,f], Pui-Kin So [c], Ting-Fan Leung [d,e], Man-sau Wong [a,b,f,g], Danyue Zhao [a,b,f,g]*

[a] *Department of Food Science and Nutrition, The Hong Kong Polytechnic University, Hong Kong, China*
[b] *Centre for Eye and Vision Research (CEVR), 17W Hong Kong Science Park, Hong Kong, China*
[c] *University Research Facility in Life Sciences, The Hong Kong Polytechnic University, Hong Kong, China*
[d] *Department of Paediatrics, The Chinese University of Hong Kong, Prince of Wales Hospital, Shatin, Hong Kong SAR, China*
[e] *Hong Kong Hub of Paediatric Excellence, The Chinese University of Hong Kong, Shatin, Hong Kong SAR, China*
[f] *Research Institute for Future Food, The Hong Kong Polytechnic University, Hong Kong, China*
[g] *Research Center for Chinese Medicine Innovation, The Hong Kong Polytechnic University, Hong Kong, China*

***Corresponding author:**

Dr. Danyue Zhao, Department of Food Science and Nutrition, The Hong Kong Polytechnic University, Hong Kong, China.
Tel.: +852 3400 8724; Fax: +852 2364 9932.
E-mail address: daisydy.zhao@polyu.edu.hk (D.Z.)

Table of Contents

Supporting Information 1
EXPERIMENTAL SECTION 3
Chemicals and Reagents 3
Instrumentation and LC-MS parameters 3
Table S1. UHPLC-HRMS parameters using the HSS T3 column at the metabolomics step. 3
Table S2. UHPLC-HRMS parameters using the BEH Amide column at the metabolomics step. 5
Table S3. UHPLC-HRMS parameters using the BEH C18 column at the lipidomics step. 6
Data Analysis 7
RESULTS AND DISCUSSION 7
Table S4. The recoveries of non-lipid small metabolites following two, three and four rounds of elution. 7
Table S5. The recoveries of non-lipid small metabolites using different elution solvents.8
Table S6. The recoveries of lipids using two solvent systems with one and two rounds of elution. 8
Table S7. The recoveries of lipids using two, three or four rounds of elution at the lipidomics step. 9
Table S8. The recoveries of lipids eluted using different solvent systems and mixing modes. 9
Table S9. Absolute recoveries of non-lipid small metabolites at the metabolomics step using different elution solvents. 10
Table S10. Absolute recoveries of representative lipid metabolites with the different solvents for eluting metabolites at the metabolomics step. 11
Table S11. Recoveries of representative metabolites present in human blood using the optimized method. 11
Table S12. Recoveries, matrix effect and repeatability assessed using isotope-labeled internal standards. 13
Table S13. The recovery (expressed as relative abundance) of annotated metabolites eluted at the metabolomics step with EMR-lipid processing compared to that without processing. 14
Table S14. The relative recovery (expressed as relative abundance) of annotated lipids at the lipidomics step with EMR-lipid processing compared to that without the processing... 21
Table S15. The high-abundance lipids recovered in the lower phase following EMR-lipid processing. 33
Table S16. The small metabolites detected in the upper phase of MTBE extracts. 35
Table S17. List of full names for non-lipid small metabolite classes reported in Figure 6A. 35
Table S18. List of full names for lipid metabolite classes reported in Figure 6B. 38
Figure S1. Pathway enrichment analysis of the annotated metabolites shared across maternal and cord blood sera. 39

EXPERIMENTAL SECTION

Chemicals and Reagents

All solvents for LC-MS analyses including acetonitrile (ACN), methanol (MeOH), isopropyl alcohol (IPA), chloroform ($CHCl_3$), methyl tert-butyl ether (MTBE) and formic acid (FA) (Macklin, Shanghai, China) were of LC-MS or HPLC grade unless otherwise specified. Ultra-pure water was produced using a Milli-Q system (Millipore, Saint-Quentin en Yvelines, France). Isotope-labeled metabolite standards (purity >98%), including Metabolomics QReSS Standard Mix and cholic acid (2,2,4,4-D4), were from Cambridge Isotope Laboratories (CIL, Andover, MA, USA). The QReSS mix was reconstituted in 50% MeOH. 4-Chloro-DL-phenylalanine was from Sigma-Aldrich (St Louis, MO, USA). 12-[[(cyclohexylamino)carbonyl]amino]- dodecanoic acid (CUDA) was from Alfa Biotechnology Co., Ltd. (Chengdu, Sichuan, China).

The standard mixture used for LC−MS method development includes creatine and cis-Aconitate (Yuanye Bio-Technology Co., Ltd., Shanghai, China); L-carnitine, citrate, epicatechin, and quercetin (Aladdin Bio-Chem Technology Co., Ltd., Shanghai, China). Adenine, adenosine, glucose-6-phosphate (G6P), uridine-monophosphate, propionyl-L-carnitine, lauroyl-L-carnitine, cholic acid, chenodeoxycholic acid, and kaempferol (Sigma-Aldrich, USA). Amino acid standards were from the Kairos Amino Acid Kit (Waters Co., Milford, MA, USA). All standards were dissolved in 80% methanol (except creatine in water) before diluted to concentrations comparable to their endogenous levels in human blood in the final working solutions.

Instrumentation and LC-MS parameters

Metabolomics methods

The instrumentation consists of an Orbitrap IQ-X Tribrid mass spectrometer (Thermo Fisher Scientific, Waltham, MA, USA) coupled to a Vanquish Horizon UHPLC system (Thermo Fisher Scientific). For nontargeted metabolomics, chromatographic separation is achieved using an ACQUITY UPLC HSS T3 column (1.8 µm, 2.1 × 100 mm; Waters Corporation, Milford, MA, USA). During separation, the column compartment is maintained at a temperature of 30 °C. Mobile phase A consisted of 0.1% formic acid in water. Mobile phase B consisted of 0.1% formic acid in ACN. The LC gradient and MS parameters are shown in **Table S1**. For very polar metabolites, chromatographic separation was achieved using an ACQUITY UPLC BEH Amide column (1.7 µm, 2.1 ×100 mm; Waters Corporation, Milford, MA, USA). During separation, the column compartment is maintained at a temperature of 40 °C. Mobile phase A consisted of 5 mM Ammouium formate and 0.1% formic acid in water. Mobile phase B consisted of 5 mM Ammouium formate, 0.1% formic acid, 95% ACN and 5% H_2O. The LC gradient and MS parameters are shown in **Table S2.** The MS parameters described for metabolomics are specific to the Thermo Orbitrap IQ-X Tribrid mass spectrometer.

Table S1. UPLC-HRMS parameters using the HSS T3 column at the metabolomics step.

LC gradient (HSS T3 column, 15 min/sample).

Time (min)	Flow (mL/min)	%B
0.0	0.3	2.0
1.0	0.3	2.0
5.0	0.3	35.0
8.0	0.3	50.0
10.0	0.3	99.0
12.0	0.3	99.0
12.1	0.3	2.0

15.0	0.3	2.0

MS parameters

Parameter	Value
Ionization mode	ESI positive
Spray voltage	+3.5 kV
Sheath gas flow rate	40
Auxiliary gas flow rate	10
Sweep gas flow rate	2
Ion transfer tube temperature	300 ℃
Vaporizer temperature	320 ℃
MS1 resolution	120,000
Scan range (m/z)	70–1200
AGC target (MS1)	Standard
Maximum Injection Time Mode (MS1)	Auto
Intensity threshold	Intensity threshold 1.0e5
Dynamic exclusion	6 s
MS^n Acquisition mode	Data-dependent
Time Between Master Scans (sec)	0.6
MS/MS resolution	MS/MS resolution 30,000
Collision Energy Type	HCD
AGC target (MS/MS)	Standard
Maximum Injection Time Mode (MS/MS)	Custom
Isolation window	1.6 m/z
Normalized collision energy (NCE)	Stepped NCE; 15, 25, 35

Parameter	Value
Ionization mode	ESI negative
Spray voltage	-2.3 kV
Sheath gas flow rate	40
Auxiliary gas flow rate	10
Sweep gas flow rate	2
Ion transfer tube temperature	300 ℃
Vaporizer temperature	320 ℃
MS1 resolution	120,000
Scan range (m/z)	70–1200
AGC target (MS1)	Standard
Maximum Injection Time Mode (MS1)	Auto
Intensity threshold	Intensity threshold 5.0e4
Dynamic exclusion	6 s
MS^n Acquisition mode	Data-dependent
Data Dependent Mode	Cycle Time
Time Between Master Scans (sec)	0.6
MS/MS resolution	MS/MS resolution 30,000
Collision Energy Type	HCD
AGC target (MS/MS)	Standard
Maximum Injection Time Mode (MS/MS)	Custom
Isolation window	1.6 m/z
Normalized collision energy (NCE)	Stepped NCE; 15, 25, 35

Table S2. UPLC-HRMS parameters using the BEH Amide column at the metabolomics step.

LC gradient (BEH Amide column, 15 min/sample)

Time (min)	Flow (mL/min)	%B
0.0	0.3	95.0
1.0	0.3	95.0
8.0	0.3	65.0
10.0	0.3	50.0
12.0	0.3	50.0
12.1	0.3	95.0
15.0	0.3	95.0

MS parameters

Parameter	Value
Ionization mode	ESI positive
Spray voltage	+3.5 kV
Sheath gas flow rate	40
Auxiliary gas flow rate	10
Sweep gas flow rate	1
Ion transfer tube temperature	300 ℃
Vaporizer temperature	320 ℃
MS1 resolution	120,000
Scan range (m/z)	60–1200
AGC target (MS1)	Standard
Maximum Injection Time Mode (MS1)	Auto
Intensity threshold	Intensity threshold 1.0e5
Dynamic exclusion	6 s
MS^n Acquisition mode	Data-dependent
Time Between Master Scans (sec)	0.6
MS/MS resolution	MS/MS resolution 30,000
Collision Energy Type	HCD
AGC target (MS/MS)	Standard
Maximum Injection Time Mode (MS/MS)	Custom
Isolation window	1.6 m/z
Normalized collision energy (NCE)	Stepped NCE; 15, 25, 35

Parameter	Value
Ionization mode	ESI negative
Spray voltage	-2.5 kV
Sheath gas flow rate	40
Auxiliary gas flow rate	10
Sweep gas flow rate	1
Ion transfer tube temperature	300 ℃
Vaporizer temperature	320 ℃
MS1 resolution	120,000
Scan range (m/z)	60–1200
AGC target (MS1)	Standard
Maximum Injection Time Mode (MS1)	Auto

Intensity threshold	Intensity threshold 5.0e4
Dynamic exclusion	6 s
MS^n Acquisition mode	Data-dependent
Time Between Master Scans (sec)	0.6
MS/MS resolution	MS/MS resolution 30,000
Collision Energy Type	HCD
AGC target (MS/MS)	Standard
Maximum Injection Time Mode (MS/MS)	Custom
Isolation window	1.6 m/z
Normalized collision energy (NCE)	Stepped NCE; 15, 25, 35

Lipidomics methods

For lipidomics, chromatographic separation was achieved using an ACQUITY UPLC BEH C18 column (1.7 µm, 2.1 × 100 mm; Waters Corporation, Milford, MA, USA). The column compartment was maintained at 50 °C. Mobile phase A consisted of 5 mM Ammouium formate, 0.1% formic acid, 40% ACN and 60% water. Mobile phase B consisted of 5 mM Ammouium formate, 0.1% formic acid, 10% ACN and 90% IPA. The LC gradient and MS parameters are shown in **Table S3**.

Table S3. UPLC-HRMS parameters using the BEH C18 column at the lipidomics step.

LC gradient using the BEH C18 column for nontargeted lipidomics (24 min/sample).

Time (min)	Flow (mL/min)	%B
0.0	0.3	40.0
2.0	0.3	40.0
2.5	0.3	58.0
18.0	0.3	99.0
20.0	0.3	99.0
20.1	0.3	40.0
24.0	0.3	40.0

MS parameters

Parameter	Value
Ionization mode	ESI positive
Spray voltage	+3.5 kV
Sheath gas flow rate	40
Auxiliary gas flow rate	10
Sweep gas flow rate	2
Ion transfer tube temperature	300 °C
Vaporizer temperature	320 °C
MS1 resolution	120,000
Scan range (m/z)	100–1500
AGC target (MS1)	Standard
Maximum Injection Time Mode (MS1)	Auto
Intensity threshold	Intensity threshold 1.0e5
Dynamic exclusion	6 s
MS^n Acquisition mode	Data-dependent
Time Between Master Scans (sec)	1.5
MS/MS resolution	7,500

Collision Energy Type	HCD
AGC target (MS/MS)	Standard
Maximum Injection Time Mode (MS/MS)	Custom
Isolation window	1.6 m/z
Normalized collision energy (NCE)	Assisted NCE; 15, 30, 40

Data Analysis

For metabolomics data, Compound Discoverer software (v. 3.3, Thermo, Waltham, USA) was used for small molecule identification based on the exact masses and fragmentation spectra. Raw data were subjected to the workflow “Untargeted Metabolomics with Statistics Detect Unknowns with ID using Online Databases” for compound annotation (mass tolerance <5 ppm). The Select Spectra node was used with open settings and a S/N threshold at 1.5. The Detect Compounds node was used with 5 ppm mass tolerance, 10,000 min. peak intensity, peak detection S/N threshold at 1.5, and compound detection of $[M+H]^{+1}$, $[M+Na]^{+1}$, $[M+NH4]^{+1}$ ions for positive mode, $[M-H]^{-1}$, $[M+FA-H]^{-1}$ and $[M+Cl]^{-1}$ ions in negative mode. The metabolites were identified on the basis of both accurate mass and fragment mass “fingerprint” spectra via searches against the spectra of compounds available in the mzCloud database (https://www.mzcloud.org) and local mzVaults databases. Compounds that were absent in mzCloud were tentatively identified using a ChemSpider search. Multiple databases, i.e., Human Metabolome Database (https://hmdb.ca/), KEGG (https://www.genome.jp/kegg/), were loaded in the search engine.

Lipidomics data were processed in LipidSearch software (v. 4.2, Thermo Fisher Scientific, San Jose, CA, USA) for lipid metabolites feature identification. The parameters were set as below. precursor tolerance, 5 ppm; product tolerance, 0.5 Da; m-score threshold, 5; Quan m/z tolerance ± 5ppm; Quan retention time (RT) range ± 0.5 min; For data alignment, isomer filter and ID quality filter were applied: adduct ions, H^+ and $NH4^+$ for ESI positive mode, and H^-, $HCOO^-$and $CH3COO^-$for ESI negative mode. The searched lipid classes include diacylglycerol (DG), triacylglycerol (TG), lysophosphatidic acid (LPA), phosphatidic acid (PA), lysophosphatidylcholine (LPC), phosphatidylcholine (PC), phosphatidylglycerol (PG), lysophosphatidylethanolamine (LPE), phosphatidylethanolamine (PE), phosphatidylinositol (PI), lysophosphatidylglycerol (LPG), lysophosphatidylinositol (LPI), lysophosphatidylserine (LPS), phosphatidylserine (PS), Ceramides (Cer), sphingomyelin (SM), cholesterol ester (ChE), monoglycerol (MG), Sitosterol Ester (SiE), Stigmasterol Ester (StE), Zymosterol Ester (ZyE), Coenzyme (Co) and fatty acid (FA). RT tolerance was 0.1 min.

Data were exported to Microsoft Excel. All detected features were filtered by the group’s coefficient of variation (CV) of integrated peak areas, according to common measures of the variability of data points, calculated as the ratio of standard deviation to mean value. Only features with a CV below 30% were considered, as a CV value above this threshold would imply highly dispersed data. The boxplots were plotted by GraphPad Prism (v. 10.0 GraphPad Software, USA). Venn diagrams were generated using R scripts.

RESULTS AND DISCUSSION

Table S4. The recoveries of non-lipid small metabolites following two, three and four rounds of elution with 80% ACN.

Class	Metabolite	2 rounds (%)		3 rounds (%)		4 rounds (%)	
		Recovery	RSD	Recovery	RSD	Recovery	RSD

**Amino acids**						
L-Valine	89.7	7.8	103.0	2.7	134.4	11.2
L-Leucine	90.8	12.3	97.4	8.3	163.2	16.9
L-Proline	51.3	3.7	55.2	8.5	71.1	5.5
L-Methionine	76.9	8.5	89.3	4.4	95.0	3.7
**Average**	77.2	7.9	86.2	6.0	115.9	9.3
**Nucleotide, purine and derivatives**						
Adenine	72.1	7.7	79.9	5.4	82.6	3.8
**Energy metabolism intermediate**						
Citrate [a]			103.3	25.2		
**Bile acids**						
Cholic acid [a]			100.2	4.6		
**Xenobiotics**						
Theobromine	103.9	1.5	109.4	3.3	111.2	2.4

[a] Most metabolites were identified in negative mode and the table only showed the recoveries using three rounds of elution in negative mode.

Table S5. The recoveries of non-lipid small metabolites using different elution solvents.

Metabolite	70% ACN (%)		80% ACN		90% ACN		100% ACN	
	Recovery	RSD	Recovery	RSD	Recovery	RSD	Recovery	RSD
**Amino acids**								
L-Leucine	95.8	11.3	102.8	7.0	100.2	2.4	99.6	3.7
L-Valine	75.8	5.2	83.5	11.1	80.3	13.3	89.0	18.4
L-Methionine	86.9	7.7	85.9	6.5	82.2	8.7	87.7	4.8
L-Asparagine	74.9	2.7	78.3	10.2	66.0	17.1	58.9	4.1
DL-Glutamine	79.8	10.2	78.6	14.7	74.5	17.0	66.3	3.0
**Average**	82.6	7.4	85.8	9.9	80.6	11.7	80.3	6.8
**Acylcarnitines**								
L-Carnitine	66.8	13.6	69.7	12.1	73.6	3.7	73.6	8.5
Propionyl-L-carnitine	127.1	6.5	119.1	5.4	117.1	2.3	108.5	6.2
Lauroyl-L-carnitine	76.1	3.4	68.3	3.7	76.9	7.0	78.1	11.8
**Nucleotides, purine and derivatives**								
Adenine	117.0	2.8	97.3	5.4	111.5	14.1	87.4	10.9
**Bile acids**								
Cholic acid	102.9	3.5	97.9	3.4	100.9	1.1	99.5	3.1
**Xenobiotics**								
Theobromine	111.7	5.4	107.9	12.3	106.2	10.5	111.3	10.9

Table S6. The recoveries of lipids using two solvent systems with one and two rounds of elution.

Lipid	C1 (%) [a]		C2 (%) [a]		M1 (%) [a]		M2 (%) [a]	
	Recovery	RSD	Recovery	RSD	Recovery	RSD	Recovery	RSD
18:1 Lyso PE	21.9	14.2	32.3	36.8	17.2	9.1	48.0	24.2

15:0-18:1 PE	14.6	50.3	69.8	22.3	32.0	15.5	70.5	6.4
15:0-18:1 PC	2.6	15.7	12.6	22.6	7.4	9.0	17.1	24.7
d18:1-18:1 SM	4.6	20.8	9.9	24.7	13.3	14.8	23.5	26.9
15:0-18:1 PG	20.4	53.7	44.7	23.4	28.3	16.7	63.0	11.7
18:1 Lyso PC	18.9	32.6	18.2	35.4	17.4	20.6	22.5	22.2

[a] Cn (n=1 or 2) refers to $CHCl_3$ system ($CHCl_3$/MeOH/H_2O; 1:2:1; v/v/v). C1 or C2 refers to one or two rounds of elution using the $CHCl_3$ solvent system. M refers to MTBE solvent system (MTBE/MeOH/H_2O; 10:3:2.5; v/v/v). M1 or M2 refers to one or two rounds of elution using the MTBE solvent system.

Table S7. The recoveries of lipids using two, three or four rounds of elution at the lipidomics step.

Lipid	2 rounds (%)		3 rounds (%)		4 rounds (%)	
	Recovery	RSD	Recovery	RSD	Recovery	RSD
18:1 Lyso PE	39.7	19.0	45.1	18.8	45.5	11.7
15:0-18:1 PE	87.6	17.7	97.2	17.1	91.2	8.6
15:0-18:1 PC	21.0	9.6	32.8	16.8	34.7	9.8
d18:1-18:1 SM	33.8	16.5	41.2	13.3	40.3	10.6
15:0-18:1 PG	67.3	19.6	77.6	18.1	60.8	2.8
18:1 Lyso PC	28.3	5.4	32.8	0.5	36.1	3.0

Table S8. The recoveries of lipids eluted using different solvent systems and mixing modes.

Lipid	System A (%) [a]		System B (%) [a]		System C (%) [a]		System D (%) [a]	
	In-well (RSD)	Out-of-well (RSD)	In-well (RSD)	Out-of-well (RSD)	In-well (RSD)	Out-of-well (RSD)	In-well (RSD)	Out-of-well (RSD)
18:1 Lyso PE	35.8 (12.4)	55.0 (5.1)	9.0 (30.8)	50.3 (10.9)	14.4 (72.3)	23.7 (17.1)	4.2 (3.3)	25.6 (11.2)
15:0-18:1 PE	52.9 (14.4)	70.7 (6.3)	20.8 (20.2)	68.8 (8.4)	14.8 (55.7)	40.3 (19.6)	28.6 (0.6)	47.8 (8.6)
15:0-18:1 PC	26.6 (8.9)	40.2 (0.1)	14.3 (8.6)	26.8 (29.7)	2.0 (20.2)	11.8 (21.7)	3.4 (8.9)	2.3 (14.4)
d18:1-18:1 SM	32.6 (4.2)	38.9 (5.5)	10.7 (5.0)	34.6 (7.5)	0.3 (0.0)	1.9 (25.1)	1.1 (4.2)	0.7 (17.2)
15:0-18:1 PG	59.5 (2.5)	83.8 (9.4)	19.5 (8.0)	75.0 (14.8)	18.8 (1.1)	26.0 (25.2)	49.4 (1.3)	62.5 (12.3)
18:1 Lyso PC	41.5 (8.9)	38.8 (4.0)	10.5 (1.3)	51.2 (10.0)	1.4 (54.2)	2.9 (3.7)	1.4 (22.7)	1.7 (17.0)

[a] System A refers to MTBE/MeOH/H_2O (10:3:2.5, v/v/v). System B refers to $CHCl_3$/MeOH/H_2O (1:2:1, v/v/v). System C refers to $CHCl_3$/MeOH/H_2O (2:1:1, v/v/v). System D refers to $CHCl_3$/MeOH/MTBE (3:4:3, v/v/v).

Table S9. Absolute recoveries of non-lipid small metabolites at the metabolomics step using different elution solvents.

Class	Metabolite	80% ACN (%)		85% ACN		90% ACN	
		Recovery	RSD	Recovery	RSD	Recovery	RSD
**Amino acids**							
Hydrophobic amino acids							
	L-Alanine	103.1	0.2	123.7	14.1	70.8	3.6
	L-Leucine	91.7	3.1	100.1	1.3	99.1	4.6
	L-Valine	113.2	4.3	106.6	3.3	97.5	13.0
	L-Proline	130.8	8.1	154.1	10.1	160.7	4.2
Hydrophilic and uncharged amino acids							
	L-Serine	54.0	3.4	53.7	4.2	74.5	10.8
	L-Asparagine	78.4	5.5	94.2	3.5	113.5	4.4
	DL-Glutamine	71.6	11.3	88.3	2.6	99.7	1.5
	DL-Homocysteine	53.9	2.6	54.5	4.2	48.7	10.8
Acidic amino acids							
	DL-Glutamic acid	67.7	5.4	71.3	5.4	89.4	4.2
	L-Aspartic acid	81.9	1.3	88.4	1.5	100.2	6.5
Basic amino acids							
	DL-Histidine	59.8	4.5	51.0	1.5	38.1	8.7
	DL-Arginine	54.3	5.9	52.9	3.5	54.8	9.3
	DL-Lysine	67.7	5.1	80.1	11.1	86.2	0.7
Amino acid derivatives							
	Creatine	113.8	5.0	109.7	5.5	112.4	4.7
**Acylcarnitines**							
	L-Carnitine	118.7	3.5	119.6	2.5	119.6	1.0
	Propionyl-L-carnitine	148.4	15.7	191.0	2.2	154.4	13.1
	Lauroyl-L-carnitine	95.4	2.9	94.6	1.3	98.2	2.9
**Nucleotide, purine and derivatives**							
	Uridine monophosphate	52.0	8.7	71.7	22.8	74.9	7.5
	Adenine	118.0	4.6	119.0	7.5	114.0	2.9
	Adenosine	109.8	16.9	147.3	0.8	134.9	13.9
**Energy metabolism intermediates**							
	Cis-Aconitate	136.4	4.0	151.4	3.1	133.8	5.0
	Citrate	77.8	7.3	69.5	10.6	72.2	2.4
**Bile acids**							

	Cholic acid	81.6	5.8	71.9	4.0	81.3	3.3
	Chenodeoxycholic acid	87.0	8.0	69.7	2.8	80.8	8.9
**Xenobiotics**							
	Epicatechin	95.0	12.7	63.2	11.9	69.0	13.8
	Quercetin	72.5	2.2	68.5	1.6	61.5	2.8
	Kaempferol	94.7	5.0	89.8	4.0	94.0	3.3
	**Average**	88.2	6.1	92.5	5.5	91.1	6.8

Table S10. Absolute recoveries of representative lipid metabolites with the different solvents for eluting metabolites at the metabolomics step.

Lipid	80% ACN (%) [a]		85% ACN (%) [a]		90% ACN (%) [a]	
	Recovery	RSD	Recovery	RSD	Recovery	RSD
18:1 Lyso PE	45.0	7.6	65.7	7.0	82.7	7.6
15:0-18:1 PE	68.4	1.8	68.7	7.5	71.7	7.0
15:0-18:1 PC	46.1	4.6	47.3	11.3	44.6	4.1
d18:1-18:1 SM	46.5	5.5	44.9	14.1	41.3	5.3
15:0-18:1 PG	40.1	0.4	37.4	23.0	42.3	4.7
18:1 Lyso PC	44.8	5.7	48.6	5.7	55.6	7.1

[a] The percentage of acetonitrile (ACN) refers to the elution solvent used at the metabolomics step. The elution solvent used at the lipidomics step was MTBE/MeOH/H_2O (10:3:2.5, v/v/v) consistently.

Table S11. Recoveries of representative metabolites present in human blood using the optimized method.

No.	Metabolite	Recovery (%)	RSD (%)
1	L-Alanine	70.8	3.6
2	L-Leucine	99.1	4.6
3	L-Valine	97.5	13.0
4	L-Proline	160.7	4.2
5	L-Serine	74.5	10.8
6	L-Asparagine	113.5	4.4
7	DL-Glutamine	99.7	1.5
8	DL-Homocysteine	48.7	10.8
9	DL-Glutamic acid	89.4	4.2
10	L-Aspartic acid	100.2	6.5
11	DL-Arginine	54.8	9.3

12	DL-Lysine	86.2	0.7
13	Creatine	112.4	4.7
14	L-Carnitine	119.6	1.0
15	Propionyl-L-carnitine	154.4	13.1
16	Lauroyl-L-carnitine	98.2	2.9
17	Uridine monophosphate	74.9	7.5
18	Adenine	114.0	2.9
19	Adenosine	134.9	13.9
20	Cis-Aconitate	133.8	5.0
21	Citrate	72.2	2.4
22	Cholic acid	81.3	3.3
23	Chenodeoxycholic acid	80.8	8.9
24	Epicatechin	69.0	13.8
25	Quercetin	61.5	2.8
26	Kaempferol	94.0	3.3
27	18:1 Lyso PE	82.7	7.6
28	15:0-18:1 PE	71.7	7.0
29	15:0-18:1 PC	44.6	4.1
30	d18:1-18:1 SM	41.3	5.3
31	15:0-18:1 PG	42.3	4.7
32	18:1 Lyso PC	55.6	7.1

Table S12. Recoveries, matrix effect and repeatability assessed using isotope-labeled internal standards.

No.	Isotope-labeled internal standard	m/z	Precursor ion	Recovery (%, n = 3)	RSD (%, n = 3)	Repeatability (RSD %, n = 6)	Matrix effect (%, n = 3)	RSD (%, n = 3)
1	L-Leucine ($^{13}C_6$)	138.1219	M+H+	120.8	3.3	3.1	104.6	2.8
2	L-Phenylalanine ($^{13}C_6$)	172.1065	M+H+	114.6	3.6	1.9	104.5	2.2
3	Vitamin B_3 ($^{13}C_6$)	129.0754	M+H+	112.2	3.1	3.0	89.9	4.5
4	L-Tryptophan ($^{13}C_{11}$)	216.1340	M+H+	114.5	3.1	2.0	103.4	2.9
5	Creatinine (D_3)	117.0850	M+H+	110.2	1.1	5.6	100.7	8.0
6	1,4-Butanediamine ($^{13}C_4$)	166.0864	M+H+	111.8	3.0	2.2	106.7	1.2
7	Thymine ($^{15}N_2$)	129.0442	M+H+	125.1	2.1	4.2	100.2	2.9
8	L-Tyrosine ($^{13}C_6$)	188.1012	M+H+	115.5	2.6	4.1	103.2	4.6
9	L-Alanine ($^{13}C_3$/^{15}N)	94.0650	M+H+	130.4	14.0	14.8	115.5	2.3
10	Cholic acid (D_4)	411.3043	M-H-	106.9	0.7	2.2	96.2	1.0
11	Hypoxanthine ($^{13}C_5$)	140.0474	M-H-	102.8	4.4	3.3	104.5	2.2
12	Guanosine ($^{15}N_5$) [a]	287.0681	$M-H^-$	74.2	39.6			

[a] Guanosine was excluded from follow-up validation experiments as it showed relatively large variation due to its low concentration in the IS mixture and weak signal (peak area ~10^4).

Table S13. The recovery (expressed as relative abundance) of annotated metabolites eluted at the metabolomics step with EMR-lipid processing compared to that without processing.

No.	Metabolite ID	Relative abundance (%)	RSD (%)	Log P
1	5-Hydroxy-2-furoic acid	75.0	20.6	-0.62
2	N,N-Dimethylacetamide	75.0	18.0	-0.49
3	Benzoic acid	75.6	3.2	1.59
4	alpha-Aminoadipic acid	75.7	20.3	-0.98
5	13S-hydroxyoctadecadienoic acid	75.9	8.8	5.04
6	Hex-2-ulose	75.9	5.4	-2.8
7	2,2,6,6-Tetramethyl-4-piperidinol	76.5	5.2	0.37
8	4-vinylphenol sulfate	76.6	5.6	0.92
9	gamma-Aminobutyric acid	77.4	6.2	-0.82
10	Pyrogallol	77.4	4.4	0.87
11	2-C-methylerythritol 4-phosphate	77.5	13.9	-0.62
12	guaiacol sulfate	78.2	5.6	0.16
13	Fomepizole	79.1	3.1	-0.09
14	alpha-Hydroxytriazolam	79.3	3.5	4.02
15	3-Methylsulfolene	79.6	4.8	-0.6
16	Glycylsarcosine	79.7	5.4	-2.5
17	4-Nitrophenol	79.8	0.6	1.44
18	N-(3-Methylbutyl)acetamide	79.8	3.0	0.85
19	Tridemorph	79.9	7.7	5.48
20	Xanthine	80.0	11.8	-1.5
21	4-tert-Octylphenol monoethoxylate	80.2	13.6	4.73
22	Quinoline	80.3	13.7	2.12
23	Indole-3-acetic acid	80.4	14.5	1.8
24	Protocatechuic acid	80.5	1.4	0.81
25	methyl 3,5-ditert-butyl-4-hydroxybenzoate	80.8	25.2	4.87
26	Lotaustralin	81.1	10.9	-0.9
27	Capsi-amide	81.2	3.1	5.2
28	Furfural	81.3	9.0	0.4
29	N-(1-Deoxy-1-fructosyl)valine	81.8	11.4	-1.29
30	Pyruvic acid	82.0	5.4	-0.68
31	Linoelaidic Acid	82.2	10.7	5.97
32	Marinobufagenin	82.6	2.2	1.25
33	Allantoin	82.8	8.9	-1.96

34	Epomediol	82.8	1.2	-0.5
35	Ethylparaben	83.2	4.0	1.8
36	Uric Acid	83.2	9.7	-2.56
37	L-(+)-Alanine	83.6	2.5	-0.9
38	Phenyl beta-D-glucopyranoside	83.6	11.5	-0.2
39	C12-Carnitine	83.7	12.9	-1.5898
40	Indole-3-propionic acid	84.0	0.9	1.49
41	HIAA	84.1	8.0	0.69
42	5-sulfooxymethylfurfural	84.4	2.2	-0.527
43	g-Guanidinobutyrate	84.4	3.8	-0.67
44	Phenyl-p-tolyl-amine	84.4	4.9	3.99
45	Ethephon	84.5	2.7	0.62
46	Vanillin 4-sulfate	84.6	4.6	-0.36
47	Butylparaben	84.9	28.3	2.71
48	Taurine	85.5	1.4	-1.72
49	3-Hydroxyoctanoic acid	85.6	2.7	1.34
50	delta-guanidinovaleric acid	85.7	1.0	-0.26
51	Cholesterol sulfate	85.8	7.0	9.46
52	3, 5-Tetradecadiencarnitine	86.1	3.5	7.192
53	Amphetamine	86.6	5.2	1.68
54	Dinotefuran	86.9	13.8	1.3
55	Sphinganine	87.0	3.7	4.52
56	Asarone	87.5	6.6	2.66
57	(2S,3S,4S,5R,6R)-6-{[(3R,5R,7R,8R,9S,10S,13R,14S)-17-{(2R)-5-[(Carboxymethyl)amino]-5-oxo-2-pentanyl}-7-hydroxy-10,13-dimethylhexadecahydro-1H-cyclopenta[a]phenanthren-3-yl]oxy}-3,4,5-trihydroxytetrahydro-2H-pyran-2-carboxylic acid	87.9	3.4	1.2
58	Metalaxyl	87.9	1.5	1.83
59	L-Palmitoylcarnitine	88.0	4.3	0.526
60	Cer(d18:1/14:0)	88.2	8.1	9.52
61	cis-5-Tetradecenoylcarnitine	88.2	2.8	-1.158
62	Bufexamac	88.3	0.7	2.17
63	Cxa-10	88.3	2.4	5.41
64	Linoleyl carnitine	88.3	2.6	0.9675
65	Propylparaben	88.3	2.6	2.29

66	3,6,9,12-tetraoxatridecan-1-ol	88.5	16.5	-0.9
67	Sulfaethidole	88.5	3.5	2.36
68	N-methylethanolamine phosphate	88.6	4.9	0.11
69	Phenylacetylglycine	88.6	8.1	1.29
70	Glycochenodeoxycholic acid	88.7	1.7	2.99
71	Desonide	88.8	2.1	0.88
72	N-Docosahexaenoyl GABA	88.9	10.3	6.68
73	Palmitoleamide	89.0	3.4	4.24
74	DL-Carnitine	89.1	2.8	-7.727
75	Taurohyocholic acid	89.3	3.3	1.77
76	DL-Tryptophan	89.4	2.7	0.32
77	Hexadecasphinganine	89.4	1.2	3.69
78	Ethanoic anhydride	89.5	5.0	-0.33
79	DL-Mevalonic acid	89.7	2.7	-0.93
80	DL-Phosphinothricin	89.7	5.6	3.29956
81	9-Decenoylcarnitine	89.8	1.4	-3.1318
82	Decanoylcarnitine	89.8	4.4	-2.6478
83	N-(2-Hydroxyethyl)tetradecanamide	90.0	2.9	4.1
84	2,6 Dimethylheptanoyl carnitine	90.2	1.5	-3.5268
85	DL-Tyrosine	90.2	0.8	0.39
86	Homovanillic acid	90.5	1.8	1.2
87	Taurochenodeoxycholic acid	90.5	1.9	2.66
88	Diethyl phthalate	90.6	3.7	2.35
89	Glucozaluzanin C	90.6	2.4	-0.35
90	D-Glucosyldihydrosphingosine	90.9	2.6	2.78
91	Athamantin	91.0	1.1	4.12
92	Uracil	91.4	7.3	-0.95
93	Trolox	91.5	2.2	3.19
94	Lucel	91.7	1.3	3.44
95	Phenylacetylglutamine	91.7	2.4	-0.27
96	CMPF	91.8	1.4	1.71
97	Tetraglyme	92.1	14.2	-0.53
98	4-Phenolsulfonic acid	92.2	7.6	0.8
99	4-Hydroxy-5-methyl-3(2H)-thiophenone	92.3	6.7	-1.46
100	Estreptoquinasa	92.3	24.1	1.5
101	Homo-L-arginine	92.5	4.6	-1.1
102	Traumatic Acid	92.6	9.1	2.68

103	Metipranolol	92.7	0.7	2.7
104	DL-Phenylalanine	92.9	4.5	0.78
105	Coumarone	93.0	1.4	1.65
106	1-Hydroxyisoquinoline	93.1	5.6	1.95
107	Phenamil	93.2	4.7	1.46
108	Imidazolone	93.4	4.1	-0.65
109	Isoprene	93.4	6.8	1.8
110	20-Oxopregn-5-en-3-yl hydrogen sulfate	93.6	2.0	3.967
111	Linoleoyl ethanolamide	93.7	5.6	5.84
112	Sulfoglycolithocholic acid	93.8	0.8	5.8276
113	1-Phenylurea	93.9	0.6	0.61
114	Selenium Sulfide	93.9	2.0	-0.387
115	4-Ethylphenylsulfonic acid	94.0	2.2	0.9
116	Pregnanediol-3-glucuronide	94.0	6.8	2.52
117	Furfuranol	94.3	0.8	0.8
118	Streptidine	94.3	4.4	-3.7
119	C8-Carnitine	94.4	1.3	-3.758
120	Hypoxanthin	94.4	3.1	0.41
121	Sinalexin	94.4	0.8	2.56
122	3-(3,4-Dimethoxyphenyl)-5-hydroxy-8-(3-hydroxy-3-methylbutyl)-4-oxo-4H-chromen-7-yl beta-D-glucopyranoside	94.6	4.7	0.7
123	Indoleacrylic acid	94.6	3.5	1.53
124	Paracetamol sulfate	94.6	5.9	-0.56
125	Hexanoylcarnitine	94.8	3.4	-4.76
126	Leu-pro	94.8	1.1	-0.1
127	5-Methoxy-3-indoleaceate	94.9	4.2	0.95
128	pC-HSL	95.0	2.1	0.67
129	4-Hydroxycinnamic acid	95.1	1.7	1.54
130	Isopropyl cyanide	95.2	4.1	1.39
131	3-Carboxy-1-(beta-D-glucopyranosyl)pyridinium	95.4	3.5	-4.34
132	Cuscohygrine	95.5	4.9	1.1
133	Leu-Val	95.5	3.6	0.57
134	Piperidine	95.6	2.1	0.6
135	Diacetyl	95.7	2.1	-0.83
136	L-(+)-Leucine	95.7	2.7	0.34
137	Phosphonic acid	95.7	2.5	-2.638

138	benzal chloride	95.9	3.7	2.91
139	N-Methoxy-1-vinyl-beta-carboline	95.9	2.1	2.78
140	Premarin	95.9	2.9	2.2
141	Theophylline	95.9	1.4	-1.3
142	Adaprolol	96.0	4.3	4.2
143	Dodecanedioic acid	96.1	5.3	2.7
144	3-Hydroxyoctanoylcarnitine	96.2	7.3	-5.1
145	Muscimol	96.2	2.2	-0.37
146	N-Acetylsphinganine	96.2	16.0	4.45
147	Isobutyryl-L-carnitine	96.3	6.3	-6.04
148	Dimethyl sulfoxide	96.4	9.2	-1.49
149	Glycoursodeoxycholic acid 3-sulfate	96.5	2.1	3.746
150	Hippuric acid	96.5	2.9	0.44
151	2-Octenoylcarnitine	96.7	3.4	-5.316
152	3,4-Dihydroxymandelic acid	96.7	0.9	0.1
153	Hexanal	96.8	1.7	1.33
154	Styrene	96.8	3.6	2.67
155	N-Oleoyl-4-aminobutyric acid	97.1	7.1	5.71
156	4-Hydroxy-5-methylfuran-3(2H)-one	97.3	0.1	-2.18
157	Indoxyl sulfate	97.3	1.7	-0.3
158	Imiprothrin	97.5	13.7	1.69
159	5-Hydroxyisouric acid	97.7	0.5	-1.26
160	Dibutyl phthalate	97.8	21.5	4.16
161	Choline	97.9	1.8	-4.3636
162	Glycocholic acid	97.9	3.3	1.89
163	3-Carboxy-4-methyl-5-pentyl-2-furanpropanoic acid	98.0	1.4	2.55
164	Dehydroepiandrosterone sulfate	98.0	0.2	3.9
165	d-Corlin	98.1	0.7	1.3
166	2-Methylthiazolidine	98.3	4.2	0.71
167	Malondialdehyde	98.6	0.9	-1.11
168	N-Acetyl-L-leucine	98.6	4.6	0.27
169	3-[2-(Hydroxymethyl)-4-methoxyphenyl]-6-methoxy-4-oxo-3,4-dihydro-1(2H)-quinazolinecarbaldehyde	98.9	0.3	1.31
170	Stachydrine	99.0	1.3	-6.719
171	p-Cresylsulfate	99.1	1.5	0.44
172	4-Dodecylphenol	99.2	11.8	6.72

173	Androsterone sulfate	99.2	1.7	3.493
174	Dimethyl fumarate	99.3	2.7	0.17
175	Urea	99.3	2.7	-1.29
176	13(S)-HpOTrE	99.4	6.8	5.1
177	Tetrahydrofuran	99.6	10.2	0.4
178	Oleoylcarnitine	99.7	2.5	1.1
179	L-Fucose	99.8	0.2	-1.52
180	Pregabalin	99.8	2.3	0.75
181	Lauryldimethylamine oxide	100.4	3.3	5.998
182	Cinnamic acid	100.5	2.3	1.93
183	Malonic acid	100.6	4.0	-0.58
184	2,5-Dihydroxybenzenesulfonic Acid	100.7	3.7	0.41
185	Isovalerylcarnitine	100.7	2.2	-5.512
186	2-Hydroxybutyric acid	100.9	1.9	-0.3
187	DL-Lactic Acid	101.3	3.4	-0.51
188	Glycerol alpha-monochlorohydrin	101.4	4.3	-0.1
189	Lethidrone	101.4	7.4	1.88
190	L-(+)-Valine	101.5	13.4	-0.1
191	cis-12-Oxophytodienoic acid	101.6	13.8	4.1
192	Propionic acid	101.8	1.4	0.35
193	N,N-Dimethyldecylamine N-oxide	101.9	0.7	4.94
194	4-Trimethylammoniobutanoic acid	102.3	4.6	-7.121
195	2,2-Bis(hydroxymethyl)propionic acid	102.8	2.1	-0.5
196	Conessine	102.8	3.4	4.8
197	(±)-(Z)-2-(5-Tetradecenyl)cyclobutanone	102.9	4.4	6.27
198	Uridine	103.2	2.1	-2.28
199	Sunitinib	103.4	0.7	2.3
200	Myristyl sulfate	103.7	7.5	4.37
201	Phytosphingosine	103.9	3.8	3.57
202	Hydrogen bromide	104.0	5.9	0.65
203	(+)-Eudesmin	104.2	14.7	2.46
204	Chloromethyl ether	104.2	1.8	1.14
205	Glycidyl oleate	104.2	8.7	6.13
206	Propionylcarnitine	104.2	1.5	-6.358
207	1,26-Hexacosanediyl (2E,2'E)bis[3-(4-hydroxy-3-methoxyphenyl)acrylate]	104.7	1.6	15.2964
208	Hexitol	104.8	3.3	-2.94

209	N,N-dimethylarginine	104.9	7.3	-0.53
210	Juniperic acid	105.0	16.5	4.57
211	12-Aminododecanoic acid	105.2	2.6	2.52
212	Cholic acid	105.6	2.0	3.4
213	Acridine	105.7	1.6	3.54
214	Elaeokanine C	105.7	6.6	1.2
215	Phenyl vinyl sulfide	106.1	10.3	3.6
216	Chlorphenesin	106.3	4.7	1.41
217	Hydroxychloroquine	106.3	7.2	2.87
218	Myxalamid A	106.6	3.6	4.72
219	3-Methylcyclopentene	106.8	1.5	2.1
220	Ethyl butylacetylaminopropionate	107.2	5.5	0.92
221	Dodecyltrimethylammonium	107.6	13.5	0.563
222	Meglumine	108.3	0.5	-2.8
223	Bis(2-ethylhexyl) amine	108.4	6.6	5.65
224	Mefenacet	108.4	2.7	3.85
225	Etoglucid	109.0	7.8	-1.22
226	5-Hydroxydecanoic acid	109.9	1.0	2.4
227	Dehydrophytosphingosine	110.3	5.8	3.25
228	Creatinine	110.6	1.7	-0.63
229	C16-Dihydroceramide	110.9	25.8	13.825
230	Stearoylethanolamide	111.5	7.2	5.67
231	N-Decanoylglycine	112.0	13.1	2.12
232	2-(Diethoxymethyl)furan	112.5	5.2	1.4
233	Glutaral	112.6	6.9	-0.75
234	N-cyclooctylurea	112.8	1.6	1.33
235	Gaboxadol	113.0	0.9	-1.62
236	Carisoprodol	113.1	7.9	1.96
237	3,5-Dinitro-2-hydroxybenzoic acid	113.9	1.2	0.75
238	4-Feruloylquinic acid	113.9	4.5	-0.49
239	Lenticin	114.2	7.0	-6.656
240	trans-Anethole	114.7	4.7	2.91
241	Ibufenac	114.9	2.1	3.19
242	Suberic acid	115.7	8.5	1.3
243	2-Methoxy-3-methylpyrazine	115.9	4.5	0.65
244	Tetralin	116.1	5.7	3.34
245	Corchorifatty acid F	116.3	24.2	2.83

246	2,5-Dimethyl-4-pyrimidinamine	116.6	16.0	1.41
247	Fluorochloridone	116.6	1.3	3.38
248	Glycolic acid	116.6	6.0	-1.1
249	4-Aminobenzoic acid	116.8	0.8	0.79
250	Stigmatellin Y	116.9	5.8	4.38
251	4-Undecylbenzenesulfonic acid	117.0	7.3	5.85
252	Embelin	117.5	4.7	1.79
253	Nonyltrimethylammonium	117.8	6.7	-1.24
254	Leu-Leu	117.9	15.7	0.92
255	Nicotinamide	117.9	9.7	-0.4
256	Metharbital	118.2	13.6	0.98
257	Azelaic acid	119.1	5.5	1.45
258	N-Undecanoylglycine	119.7	5.1	2.54
259	N-Acetylvaline	121.5	8.7	-0.8
260	Corey PG-lactone diol	121.6	4.1	1.45
261	Paspaline	122.4	8.3	4.47
262	1,3,5-Trimethyl-4-[2-(1,3,5-trimethyl-1H-pyrazol-4-yl)diaz-1-enyl]-1H-pyrazole	122.5	25.4	-1.57
263	Undecanedioic acid	122.5	10.2	2.28
264	Bargustanine	123.8	13.0	3.17
265	Erucamide	123.9	2.0	7.31
266	Pivagabine	123.9	12.4	1.04
267	Coenzyme Q2	124.1	6.6	1.32
268	Indole-3-acetaldehyde	124.2	7.7	0.81
269	Indospicine	124.3	6.2	-0.97
270	10Z-Heptadecenoic acid	124.9	7.4	5.87

Table S14. The relative recovery (expressed as relative abundance) of annotated lipids at the lipidomics step with EMR-lipid processing compared to that without the processing.

No.	Lipid	Class	Relative abundance (%)	RSD (%)
1	TG(O-18:1_16:0_18:1)	TG	75.1	6.1
2	DG(O-32:2_16:0)	DG	75.2	8.7
3	PC(O-25:1_21:4)	PC	75.2	24.9
4	SM(d44:6)	SM	75.3	2.8
5	PC(18:0_22:5)	PC	75.4	8.5
6	LPC(28:0)	LPC	75.4	11.5
7	TG(17:0_18:0_18:1)	TG	75.5	2.7
8	PC(43:8)	PC	75.5	15.2
9	TG(18:0_18:1_22:5)	TG	75.6	11.9

10	PC(38:4CHO)	PC	75.6	7.5
11	TG(16:0_18:1_10:2CHO)	TG	75.6	16.9
12	PC(37:7)	PC	75.6	10.4
13	TG(P-5:0_18:1_13:0)	TG	75.6	7.4
14	TG(16:0_18:1_18:2CHO)	TG	75.6	20.8
15	PC(O-37:8)	PC	75.7	1.7
16	PC(24:5_21:6)	PC	75.9	15.1
17	PC(16:0_28:8)	PC	76.0	16.6
18	ChE(16:1)	ChE	76.1	18.7
19	PC(O-38:7)	PC	76.1	8.3
20	PC(O-18:0)	PC	76.2	23.0
21	TG(20:1_16:0_18:0)	TG	76.3	7.3
22	PC(41:4COOH)	PC	76.4	4.8
23	DG(16:0_18:1)	DG	76.5	9.8
24	PC(O-37:6)	PC	76.6	8.3
25	PC(36:4)	PC	76.6	11.5
26	PC(15:0_20:4)	PC	76.6	11.5
27	Cer(d12:0_16:0)	Cer	76.6	18.7
28	PC(P-41:12)	PC	76.7	14.0
29	PC(O-32:2)	PC	76.8	10.5
30	PC(18:1_22:6)	PC	76.9	5.9
31	PC(36:0)	PC	76.9	22.1
32	PC(44:4)	PC	76.9	17.8
33	TG(O-16:0_16:0_18:1)	TG	76.9	3.5
34	WE(O-27:5_20:5)	WE	77.0	5.7
35	PC(O-35:4)	PC	77.1	13.4
36	PE(P-16:0_22:4)	PE	77.1	3.6
37	PC(34:0CHO)	PC	77.1	9.5
38	PC(39:7COOH)	PC	77.2	14.9
39	TG(16:0_18:1_20:1)	TG	77.2	2.3
40	PC(18:0_18:2)	PC	77.2	8.6
41	PC(36:3)	PC	77.4	8.2
42	TG(9:0COOH_16:0_18:0)	TG	77.4	22.0
43	PC(38:7)	PC	77.5	11.8
44	SM(d36:0)	SM	77.6	19.0
45	TG(27:1_17:0)	TG	77.7	8.0
46	d5-DG(14:0_16:0)	d5-DG	77.7	1.6
47	PE(P-16:0_22:6)	PE	77.7	10.3
48	PC(O-34:3)	PC	77.8	4.1
49	TG(16:0_16:0_16:3)	TG	77.8	10.7
50	PC(33:6)	PC	77.8	10.6
51	TG(20:0_18:1_18:2)	TG	77.8	2.1
52	DG(16:1_18:1)	DG	77.8	6.2
53	PC(O-13:1_22:6)	PC	77.9	7.4
54	PEt(O-15:1_19:2)	PEt	77.9	4.1
55	PC(20:4_16:0)	PC	77.9	17.0
56	SM(d18:2_25:3)	SM	77.9	13.7
57	SM(d38:2)	SM	78.1	7.9
58	TG(18:2_18:2_22:5)	TG	78.2	20.6
59	SM(d42:5)	SM	78.3	8.5

60	TG(12:0_18:2_22:6)	TG	78.4	16.4
61	TG(18:1_22:1_18:2)	TG	78.4	1.0
62	PC(34:1)	PC	78.4	9.8
63	TG(15:0_18:1_20:4)	TG	78.4	10.4
64	Cer(d14:1_2:0)	Cer	78.4	17.3
65	SM(d18:1_26:4)	SM	78.5	13.9
66	PC(36:2)	PC	78.5	19.6
67	PC(O-36:5)	PC	78.6	5.0
68	TG(16:0_17:0_18:1)	TG	78.6	5.0
69	AEA(12:0)	AEA	78.6	22.6
70	Cer(d17:1_34:4)	Cer	78.6	18.9
71	PC(O-36:6)	PC	78.7	11.0
72	LPC(30:0)	LPC	78.8	11.8
73	PC(19:4_19:3)	PC	78.8	5.6
74	TG(16:0_18:3_22:6)	TG	78.9	24.9
75	PC(42:8)	PC	78.9	6.4
76	PC(O-15:2_20:4)	PC	79.0	1.6
77	PC(O-38:5)	PC	79.0	10.3
78	PC(38:6CHO)	PC	79.0	3.6
79	TG(21:1_16:0)	TG	79.0	9.7
80	TG(O-16:0_16:0_18:2)	TG	79.0	11.3
81	PC(21:1CHO)	PC	79.1	8.3
82	PE(O-40:7)	PE	79.2	9.8
83	PC(16:0_14:0)	PC	79.3	5.8
84	PE(18:0_20:4)	PE	79.4	5.5
85	TG(13:0COOH_13:0)	TG	79.5	16.4
86	TG(16:0_21:0_18:1)	TG	79.5	8.6
87	MG(O-18:3)	MG	79.5	16.2
88	TG(18:0_18:1_20:4)	TG	79.5	7.8
89	SM(d42:1)	SM	79.5	19.7
90	PC(34:0)	PC	79.6	18.2
91	TG(22:1)	TG	79.6	12.9
92	PE(18:4COOH_28:2)	PE	79.7	11.3
93	Cer(t18:0_15:1)	Cer	79.7	8.2
94	PC(O-16:1_14:0)	PC	79.7	7.0
95	AcHexCm()	AcHexCm	79.7	11.9
96	PE(6:1CHO_16:0)	PE	79.7	7.5
97	PFAA(13:0)	PFAA	79.8	25.2
98	TG(26:0_20:5)	TG	79.8	14.4
99	SM(d39:0)	SM	79.9	12.2
100	PC(O-37:4)	PC	79.9	8.9
101	LPE(16:0)	LPE	79.9	1.0
102	PC(O-37:7)	PC	80.1	9.3
103	AcCa(26:0)	AcCa	80.3	8.3
104	SM(d34:0)	SM	80.4	15.3
105	PC(36:1CHO)	PC	80.7	10.0
106	PC(18:0_20:4)	PC	80.7	7.1
107	PC(O-44:10)	PC	80.9	6.8
108	TG(16:0_18:1CHO_18:2)	TG	80.9	17.4
109	SM(d41:5)	SM	80.9	9.6

110	PFAA(19:0)	PFAA	81.0	5.8
111	TG(16:0_18:1_18:3)	TG	81.0	4.9
112	Cer(m18:3_24:0)	Cer	81.2	12.4
113	TG(18:0_18:1_20:3)	TG	81.2	8.2
114	TG(O-16:0_18:1_18:2)	TG	81.2	8.2
115	PC(35:7)	PC	81.3	6.6
116	PE(22:6_17:0)	PE	81.3	15.0
117	PC(18:2_17:2COOH)	PC	81.4	3.3
118	TG(16:0_18:2_21:5)	TG	81.4	7.1
119	Cer(d16:0_16:0)	Cer	81.4	7.4
120	TG(17:0_18:1_20:1)	TG	81.4	2.9
121	PC(O-26:0_20:6)	PC	81.5	11.0
122	ChE(18:1)	ChE	81.5	6.8
123	PC(36:8)	PC	81.5	17.5
124	TG(18:1_19:1_18:2)	TG	81.6	9.0
125	DG(O-17:1_8:0)	DG	81.6	20.9
126	PEt(O-15:1_17:0)	PEt	81.6	18.7
127	PC(35:1CHO)	PC	81.6	13.0
128	TG(13:0_16:0_16:0)	TG	81.7	19.7
129	AEA(18:0)	AEA	81.7	3.0
130	Cer(d18:2_24:0)	Cer	81.7	15.1
131	TG(16:0_16:0CHO_18:1)	TG	81.7	10.1
132	DG(O-18:4_18:2)	DG	81.7	10.2
133	PC(O-30:6_8:0)	PC	81.8	11.2
134	DG(P-7:0_16:0)	DG	81.8	10.1
135	SM(t18:0_16:1)	SM	81.8	16.0
136	TG(16:0_16:0_17:0)	TG	81.9	11.7
137	PI(35:2)	PI	82.0	15.0
138	Cer(d17:0_16:0)	Cer	82.2	12.7
139	PE(20:4_20:4)	PE	82.2	9.0
140	DG(O-15:1_10:0)	DG	82.2	8.0
141	PC(18:2_16:0)	PC	82.5	5.7
142	PC(43:6COOH)	PC	82.5	12.2
143	TG(O-13:0_16:0_3:0)	TG	82.6	12.2
144	PC(23:7_16:0)	PC	82.6	7.5
145	PE(P-18:1_20:4)	PE	82.7	5.3
146	TG(16:0_17:0_18:2)	TG	82.7	5.6
147	PC(P-15:2_20:5)	PC	82.7	4.6
148	TG(30:0_22:6)	TG	82.7	26.6
149	PE(P-16:0_20:4)	PE	82.8	6.1
150	SM(d43:6)	SM	82.9	13.2
151	PC(40:9)	PC	83.0	9.6
152	TG(18:2_20:5_22:6)	TG	83.0	5.6
153	PC(P-18:4_18:0)	PC	83.1	5.3
154	TG(P-5:0_16:1_2:0)	TG	83.1	17.5
155	TG(18:1_20:4_22:5)	TG	83.2	8.1
156	PE(O-20:2_18:2)	PE	83.2	17.4
157	SM(d35:0)	SM	83.2	10.6
158	TG(15:0_16:0_18:0)	TG	83.2	6.2
159	TG(15:0_18:1_22:6)	TG	83.3	6.3

160	PC(16:0_16:0)	PC	83.3	10.1
161	PFAA(12:1)	PFAA	83.3	14.9
162	TG(16:0_18:1_21:5)	TG	83.4	16.0
163	PE(36:4)	PE	83.4	19.7
164	TG(18:2_20:4_22:6)	TG	83.4	9.6
165	SM(d35:4)	SM	83.4	9.9
166	TG(O-14:2_4:0_18:2)	TG	83.4	3.1
167	DG(16:0_18:0)	DG	83.5	7.9
168	Cer(m18:0_18:0)	Cer	83.5	7.2
169	TG(15:0_16:0_22:6)	TG	83.5	12.2
170	PE(18:1_20:4)	PE	83.5	6.5
171	SM(d40:0)	SM	83.6	21.9
172	PC(21:0COOH)	PC	83.6	25.9
173	TG(16:0_20:5_22:6)	TG	83.7	9.5
174	TG(18:0_20:2_20:4)	TG	83.8	8.7
175	ChE(20:5)	ChE	83.8	9.8
176	TG(18:1_18:1_19:1)	TG	83.8	4.6
177	TG(16:1_2:0_5:0)	TG	83.8	6.8
178	PE(O-36:4)	PE	83.8	19.4
179	TG(15:0_18:2_22:6)	TG	83.8	14.8
180	TG(18:0_18:1_24:5)	TG	83.9	9.0
181	TG(18:1_18:1_24:5)	TG	83.9	8.6
182	TG(6:0_16:0_18:2)	TG	84.0	14.7
183	Co(Q10)	Co	84.0	7.0
184	TG(18:0_18:2_20:4)	TG	84.0	15.6
185	SM(d38:4)	SM	84.1	17.3
186	SM(d18:2_22:1)	SM	84.2	14.6
187	Cer(m18:1_22:0)	Cer	84.2	7.3
188	TG(17:0_18:1_18:1)	TG	84.2	5.9
189	PC(18:2_18:2)	PC	84.3	7.0
190	TG(18:1_16:0_18:3)	TG	84.3	4.2
191	PC(22:6_15:0)	PC	84.3	9.5
192	ChE(18:3)	ChE	84.4	8.1
193	PC(38:4)	PC	84.4	9.0
194	TG(16:0_11:1_18:1)	TG	84.4	9.5
195	TG(12:0_12:0_18:1)	TG	84.4	14.7
196	TG(14:0_15:1_18:2)	TG	84.5	19.7
197	Hex2Cer(d18:1_16:0)	Hex2Cer	84.6	17.8
198	SM(t36:4)	SM	84.7	9.5
199	TG(14:0_16:0_18:1)	TG	84.8	3.9
200	PC(16:0_18:1)	PC	84.8	6.2
201	TG(22:7_18:2_20:4)	TG	84.8	9.5
202	SM(d18:1_24:1)	SM	84.8	17.0
203	TG(18:1_18:1_18:2CHO)	TG	84.9	12.2
204	PC(33:3)	PC	84.9	7.0
205	PC(13:0_22:5)	PC	84.9	6.1
206	TG(14:0_16:0_18:3)	TG	84.9	12.7
207	TG(14:0_16:0_18:2)	TG	85.0	9.1
208	TG(15:0_16:1_18:3)	TG	85.0	12.3
209	WE(O-27:5_18:1)	WE	85.0	9.5

210	TG(16:0_15:1_18:2)	TG	85.0	10.5
211	Cer(m18:3_22:0)	Cer	85.1	17.0
212	WE(O-27:5_22:6)	WE	85.1	11.3
213	PE(18:0_18:1)	PE	85.2	7.1
214	Cer(t11:0_16:0)	Cer	85.2	7.3
215	DG(O-15:1_16:1)	DG	85.3	7.7
216	TG(16:0_18:0_22:0)	TG	85.3	1.4
217	TG(15:1_3:0_3:0)	TG	85.3	10.6
218	PE(18:1_18:2)	PE	85.3	7.1
219	TG(18:1_18:2_24:6)	TG	85.3	3.7
220	TG(18:2_18:3_22:6)	TG	85.6	11.4
221	TG(16:0_18:1_22:4)	TG	85.6	7.4
222	PC(18:2_15:0)	PC	85.7	4.5
223	TG(12:0_12:0_18:2)	TG	85.7	17.9
224	DG(O-15:1_18:0)	DG	85.7	13.6
225	WE(O-27:5_20:3)	WE	85.7	4.0
226	DG(16:1_20:4)	DG	85.7	5.7
227	TG(18:2_20:4_22:5)	TG	85.7	14.3
228	TG(14:0_18:2_20:5)	TG	85.8	13.0
229	TG(18:1_22:5_22:6)	TG	85.9	8.7
230	Hex2Cer(d18:2_16:0)	Hex2Cer	85.9	4.8
231	SM(t40:1)	SM	86.0	11.4
232	TG(12:0_18:2_20:4)	TG	86.1	9.8
233	TG(18:1_20:3_22:6)	TG	86.1	15.8
234	DG(15:0_21:2)	DG	86.1	10.8
235	DG(16:0_20:3)	DG	86.1	4.7
236	Cer(m16:0_18:0)	Cer	86.1	10.0
237	PC(O-35:1)	PC	86.2	14.3
238	DG(O-18:2_18:0)	DG	86.3	13.9
239	SM(d39:4)	SM	86.4	4.5
240	TG(16:2_18:2_18:3)	TG	86.4	7.7
241	WE(O-27:5_20:4)	WE	86.5	10.9
242	TG(6:0_16:0_18:1)	TG	86.5	14.4
243	Cer(d15:1_12:0)	Cer	86.6	8.5
244	SM(d35:2)	SM	86.6	7.3
245	Hex1Cer(d18:1_16:0)	Hex1Cer	86.7	3.9
246	PC(O-15:2_20:3)	PC	86.7	5.4
247	DG(O-14:0_11:1)	DG	86.8	22.1
248	PC(21:0_21:2)	PC	86.8	17.6
249	PC(O-42:9)	PC	86.9	5.1
250	TG(16:0_18:1_24:4)	TG	87.0	4.8
251	Cer(t20:1_18:0)	Cer	87.0	4.6
252	TG(14:0_18:2_22:6)	TG	87.1	8.1
253	TG(18:1_18:1_22:5)	TG	87.1	19.9
254	TG(4:1CHO_16:0_16:0)	TG	87.1	9.5
255	TG(15:0_18:2_18:3)	TG	87.2	7.7
256	TG(12:0_16:1_18:1)	TG	87.2	13.1
257	TG(15:0_18:1_18:3)	TG	87.2	8.5
258	TG(16:0_16:0_18:2)	TG	87.3	7.1
259	DG(O-17:1_17:0)	DG	87.3	5.2

260	AEA(13:0)	AEA	87.4	17.1
261	DG(18:0_20:4)	DG	87.4	15.6
262	SPH(d15:1)	SPH	87.5	20.1
263	Cer(d18:0_22:0)	Cer	87.5	4.5
264	TG(P-7:0_27:0)	TG	87.5	10.5
265	TG(16:0_11:1_18:2)	TG	87.6	20.0
266	PC(O-40:1)	PC	87.7	14.2
267	PC(17:0COOH_19:1)	PC	87.8	12.5
268	TG(18:1_18:1_18:1)	TG	87.9	10.0
269	SM(d31:0)	SM	87.9	5.6
270	TG(4:0_8:0_11:1)	TG	88.0	14.1
271	LPC(22:0)	LPC	88.0	24.4
272	Cer(m18:2_20:0)	Cer	88.0	20.0
273	DG(O-15:1_17:0)	DG	88.1	4.6
274	PE(36:2)	PE	88.1	12.8
275	TG(14:0_15:0_16:1)	TG	88.1	20.8
276	TG(18:1_18:2_24:5)	TG	88.2	14.8
277	TG(17:1_18:2_18:2)	TG	88.2	9.7
278	DG(18:0_18:0)	DG	88.3	4.8
279	TG(17:1_18:2_18:3)	TG	88.4	18.8
280	PC(38:9)	PC	88.4	4.6
281	Cer(m18:1_24:0)	Cer	88.4	15.0
282	SM(d44:7)	SM	88.4	7.0
283	TG(16:0_24:5_22:6)	TG	88.4	12.2
284	PC(O-13:1_22:5)	PC	88.5	6.7
285	TG(18:1_22:6_22:6)	TG	88.5	8.5
286	TG(12:0_16:1_18:3)	TG	88.5	23.6
287	TG(26:0COOH)	TG	88.6	17.0
288	PFAA(12:0)	PFAA	88.6	13.3
289	SM(t18:0_24:2)	SM	88.6	3.0
290	TG(10:3_16:0_16:0)	TG	88.6	14.0
291	TG(11:0CHO_13:0)	TG	88.7	10.9
292	TG(17:1_18:1_18:2)	TG	88.7	15.4
293	TG(12:0_16:1_18:2)	TG	88.8	13.2
294	PC(16:1_21:4COOH)	PC	88.8	7.2
295	SM(d18:2_24:2)	SM	89.0	13.0
296	TG(16:1_18:3_22:6)	TG	89.0	12.1
297	TG(18:2_22:6_22:6)	TG	89.2	12.7
298	TG(25:3)	TG	89.2	12.1
299	TG(18:1CHO_16:0_18:1)	TG	89.3	14.4
300	TG(31:8COOH)	TG	89.3	4.7
301	ChE(22:5)	ChE	89.4	16.2
302	PI(16:0_18:2)	PI	89.5	23.6
303	PC(19:1CHO)	PC	89.6	5.0
304	TG(18:2_18:3_18:3)	TG	89.6	10.1
305	PE(16:0_20:4)	PE	89.7	7.5
306	PC(O-41:10)	PC	89.7	14.7
307	TG(16:0_20:4_22:6)	TG	89.7	14.0
308	TG(10:0_12:0_14:0)	TG	89.8	15.2
309	Cer(t18:1_18:0)	Cer	89.8	17.4

310	TG(12:0_14:0_14:0)	TG	89.9	10.3
311	TG(12:1_3:0_8:0)	TG	90.0	16.3
312	PC(36:3COOH)	PC	90.0	7.5
313	TG(12:0_18:2_18:3)	TG	90.0	13.8
314	TG(26:7)	TG	90.1	6.0
315	TG(4:0_16:0_16:0)	TG	90.2	13.2
316	PE(O-38:6)	PE	90.2	16.1
317	TG(16:0_16:0_22:5)	TG	90.3	2.6
318	PC(18:0_20:5)	PC	90.4	8.4
319	TG(12:0_14:0_18:2)	TG	90.5	8.9
320	TG(16:0_18:0_19:0)	TG	90.5	3.8
321	TG(14:0_15:0_18:1)	TG	90.5	7.5
322	TG(16:0_18:2_18:2CHO)	TG	90.6	11.0
323	PE(O-16:0_18:2)	PE	90.6	10.2
324	Cer(d18:0_26:4)	Cer	90.6	14.4
325	DG(O-15:1_14:0)	DG	90.7	5.0
326	PC(18:3_19:2COOH)	PC	90.7	9.8
327	SM(d42:4)	SM	90.8	11.5
328	Hex1Cer(d18:1_24:2)	Hex1Cer	90.8	15.7
329	Cer(t19:0_16:0)	Cer	90.8	6.7
330	TG(16:1_14:2_18:2)	TG	91.0	10.1
331	PC(16:0_18:2)	PC	91.0	5.7
332	Cer(m18:3_23:0)	Cer	91.0	6.2
333	DG(O-18:2_20:0)	DG	91.1	7.6
334	TG(O-13:0_18:3_3:0)	TG	91.2	11.0
335	DG(16:0_18:3)	DG	91.2	8.8
336	PFAA(18:3)	PFAA	91.3	24.5
337	TG(18:0_20:4_22:6)	TG	91.3	11.4
338	TG(15:1_8:0)	TG	91.3	16.3
339	TG(18:3_15:1_18:1)	TG	91.3	7.5
340	SM(d36:1)	SM	91.3	11.5
341	PC(33:0)	PC	91.4	14.8
342	TG(14:0_16:0_18:0)	TG	91.5	6.6
343	SM(d18:1_26:3)	SM	91.5	16.2
344	PC(O-46:5)	PC	91.5	16.1
345	DG(16:0_16:0)	DG	91.5	11.3
346	Cer(d16:0_2:0)	Cer	91.5	26.2
347	PE(20:4_18:0)	PE	91.5	13.0
348	PE(16:0_18:2)	PE	91.7	10.9
349	PC(15:1_17:0)	PC	91.7	7.1
350	PE(20:4_17:0)	PE	91.9	5.8
351	PC(O-39:8)	PC	91.9	3.4
352	DG(16:0_20:1)	DG	92.0	9.0
353	TG(16:0_22:6_22:6)	TG	92.1	12.7
354	PC(O-16:2_21:2)	PC	92.1	7.2
355	PC(21:3CHO)	PC	92.2	19.5
356	SM(d38:3)	SM	92.2	12.9
357	DG(18:0_20:0)	DG	92.3	7.0
358	TG(17:1_18:1_22:6)	TG	92.3	8.5
359	TG(16:0_22:5_22:6)	TG	92.3	9.5

360	Cer(d17:0_12:0)	Cer	92.3	5.3
361	SM(d42:0)	SM	92.4	8.7
362	SM(d16:1_26:4)	SM	92.5	4.3
363	TG(18:1_18:0_18:2)	TG	92.5	18.9
364	DG(P-6:0_7:0)	DG	92.6	0.6
365	PE(P-18:2_18:2)	PE	92.6	5.4
366	Cer(m14:0_18:0)	Cer	92.6	4.1
367	PG(P-34:2_16:0)	PG	92.8	15.9
368	TG(14:1_18:2_18:3)	TG	92.8	29.5
369	TG(12:0_14:0_18:3)	TG	92.9	20.5
370	SM(d41:0)	SM	92.9	18.4
371	SM(t40:0)	SM	93.0	21.6
372	PC(25:0COOH)	PC	93.3	15.6
373	PC(43:6CHO)	PC	93.4	16.8
374	TG(18:1_18:2_22:5)	TG	93.5	14.3
375	TG(14:0_16:0_22:6)	TG	93.6	12.4
376	TG(15:0_16:0_18:3)	TG	93.6	13.3
377	Cer(d16:0_18:0)	Cer	93.7	14.7
378	DG(14:0_20:4)	DG	93.8	3.7
379	TG(2:0_18:0_18:0)	TG	93.9	14.8
380	PC(15:0_22:6)	PC	93.9	5.0
381	PE(18:0_22:6)	PE	93.9	5.5
382	PC(20:4_15:1)	PC	94.1	14.9
383	TG(18:2_18:1_18:2CHO)	TG	94.2	12.5
384	TG(12:0_16:0_20:4)	TG	94.2	9.5
385	DG(P-6:0_12:0)	DG	94.2	17.9
386	DG(O-19:1)	DG	94.4	27.1
387	TG(28:1_18:3)	TG	94.4	15.7
388	SM(d18:2_24:1)	SM	94.4	20.7
389	TG(17:2_19:0_19:0)	TG	94.5	18.2
390	TG(24:0CHO)	TG	94.7	9.0
391	PE(O-18:0)	PE	94.8	5.2
392	TG(17:0_18:1_22:6)	TG	94.9	18.7
393	LPE(18:0)	LPE	95.1	3.2
394	Cer(m18:2_24:0)	Cer	95.3	17.2
395	PC(27:0COOH)	PC	95.3	13.8
396	TG(19:1_16:0)	TG	95.3	2.0
397	TG(18:1_18:1_21:1)	TG	95.3	8.4
398	Cer(m18:2_18:0)	Cer	95.5	10.7
399	DG(O-25:1)	DG	95.7	3.6
400	PC(38:8)	PC	95.8	10.7
401	TG(17:0_18:0_18:0)	TG	95.9	4.1
402	TG(18:0_18:1_18:1)	TG	96.0	11.0
403	Cer(t20:1_16:0)	Cer	96.0	4.5
404	PC(O-18:4_16:0)	PC	96.2	10.1
405	DG(18:1_18:0)	DG	96.4	16.8
406	TG(18:2_18:3_20:4)	TG	96.5	22.0
407	PE(O-18:2_18:2)	PE	96.6	7.3
408	TG(O-15:0_18:0_3:0)	TG	96.6	5.8
409	Cer(t14:1_16:0)	Cer	96.6	19.5

410	Cer(d18:2_24:1)	Cer	96.7	7.9
411	TG(14:0_15:0_18:2)	TG	96.7	10.0
412	TG(16:0_18:1_18:1CHO)	TG	96.8	18.0
413	PE(P-18:1_18:2)	PE	96.9	6.4
414	Cer(d18:0_23:0)	Cer	97.0	14.1
415	Cer(d17:0_17:0)	Cer	97.4	8.0
416	TG(16:0_18:0_20:0)	TG	97.7	4.5
417	SM(t34:1)	SM	98.0	15.5
418	PC(20:4CHO_16:0)	PC	98.1	15.0
419	TG(18:2_16:0_15:2)	TG	98.3	16.4
420	PC(O-43:12)	PC	98.4	4.2
421	TG(45:6_20:0CHO)	TG	98.5	8.6
422	PC(P-39:10)	PC	98.5	10.5
423	Cer(m18:2_23:0)	Cer	98.7	13.8
424	TG(14:0_16:0_17:1)	TG	98.9	12.6
425	DG(O-15:1_8:0)	DG	98.9	8.5
426	DG(16:1_16:0)	DG	98.9	4.6
427	TG(17:0COOH_16:0_18:2)	TG	99.1	6.2
428	TG(16:0_16:0_18:0)	TG	99.1	4.5
429	PE(P-20:2_20:4)	PE	99.2	15.1
430	Cer(m18:3_24:1)	Cer	99.2	9.9
431	SPH(t15:2)	SPH	99.2	28.0
432	SM(d44:5)	SM	99.3	13.7
433	PC(40:7)	PC	99.3	12.3
434	TG(18:0_18:0_20:0)	TG	99.4	10.6
435	TG(16:0_17:0_18:0)	TG	99.7	6.9
436	PC(31:3)	PC	99.7	10.3
437	PE(20:4_18:2)	PE	99.9	28.5
438	Hex1Cer(d18:1_23:0)	Hex1Cer	99.9	13.7
439	PE(16:0_18:1)	PE	100.0	10.7
440	PC(P-16:3_20:1)	PC	100.0	6.6
441	TG(48:9_19:0)	TG	100.1	5.2
442	PE(37:3)	PE	100.3	16.6
443	Cer(d18:2_22:0)	Cer	100.3	6.7
444	PE(16:0_18:3)	PE	100.3	4.9
445	TG(P-7:0_16:0)	TG	100.4	9.1
446	TG(12:0_14:0_16:0)	TG	100.4	5.8
447	PE(34:1)	PE	100.7	18.3
448	PC(20:3_16:2)	PC	100.8	8.1
449	PC(29:2)	PC	100.8	7.8
450	TG(20:0COOH_3:0_3:0)	TG	100.8	8.0
451	DG(O-18:2_18:2)	DG	100.9	5.3
452	PC(P-38:5)	PC	101.0	9.7
453	PC(36:3CHO)	PC	101.0	9.3
454	Cer(d18:0_24:0)	Cer	101.0	12.9
455	PI(38:4)	PI	101.0	10.4
456	PE(P-20:0_18:2)	PE	101.1	18.0
457	PE(P-18:1_18:1)	PE	101.1	13.3
458	TG(2:0_16:0_16:0)	TG	101.2	8.4
459	PFAA(25:1)	PFAA	101.5	12.1

460	TG(12:0_12:0_14:0)	TG	101.7	16.8
461	Cer(d13:1_16:0)	Cer	101.8	3.5
462	TG(16:0_18:0_18:1)	TG	101.9	4.2
463	TG(10:0COOH_3:0_13:0)	TG	101.9	9.5
464	DG(O-15:1_12:0)	DG	102.0	14.4
465	PC(37:6)	PC	102.0	15.7
466	TG(16:0_18:0_18:0)	TG	102.1	6.1
467	PE(P-18:2_20:4)	PE	102.1	10.3
468	DG(18:1_16:0)	DG	102.2	11.1
469	SM(d40:4)	SM	102.3	6.6
470	DG(O-17:1_18:0)	DG	102.3	2.4
471	TG(31:1_14:0)	TG	102.4	4.7
472	PC(O-15:2_22:5)	PC	102.5	15.0
473	TG(14:0_16:0_16:0)	TG	102.5	10.3
474	TG(15:0_16:0_18:1)	TG	102.6	7.5
475	Cer(d20:0_18:0)	Cer	102.8	4.7
476	TG(29:4_19:0_17:3)	TG	102.9	8.4
477	TG(14:0_15:0_16:0)	TG	103.2	8.5
478	PC(O-35:6)	PC	103.3	8.2
479	Cer(t20:1_20:3)	Cer	103.3	5.8
480	PC(20:5_18:1)	PC	103.4	3.6
481	DG(P-16:3_23:5)	DG	103.6	2.5
482	PC(O-38:8)	PC	103.7	11.2
483	PC(O-17:1_22:6)	PC	103.8	25.9
484	PC(36:4CHO)	PC	103.8	13.4
485	PC(P-37:9)	PC	104.0	5.1
486	DG(O-16:1_18:2)	DG	104.0	15.5
487	Cer(d16:1_2:0)	Cer	104.1	9.8
488	TG(18:0_18:1_22:6)	TG	104.3	15.3
489	TG(16:0_16:1_20:5)	TG	104.3	3.2
490	TG(16:1_18:2_18:3)	TG	104.5	11.3
491	PE(P-16:0_18:2)	PE	104.6	1.8
492	PFAA(22:2)	PFAA	104.6	4.7
493	TG(25:2_22:1COOH)	TG	104.8	3.4
494	TG(23:0_18:0COOH)	TG	104.9	10.2
495	PC(33:4)	PC	104.9	2.5
496	TG(21:1_18:3)	TG	105.0	6.2
497	PC(33:5)	PC	105.3	9.7
498	PC(O-40:8)	PC	105.4	5.1
499	TG(10:0_14:0_16:0)	TG	105.8	10.9
500	Ch()	Ch	105.9	6.4
501	PE(20:5_16:0)	PE	106.0	9.2
502	Hex1Cer(d18:1_24:0)	Hex1Cer	106.0	17.6
503	DG(P-6:0_16:0)	DG	106.0	10.8
504	TG(14:0_14:0_16:0)	TG	106.2	10.9
505	PC(38:6)	PC	106.5	23.6
506	DG(O-18:2_20:5)	DG	106.5	9.1
507	Ch-D7()	Ch-D7	106.8	16.4
508	Cer(d17:1_38:7)	Cer	107.0	22.6
509	MG(18:2)	MG	107.0	9.3

510	PEt(17:2_23:0)	PEt	107.5	5.7
511	TG(O-16:1_16:0_2:0)	TG	107.6	2.4
512	Cer(t16:1_18:0)	Cer	107.8	4.1
513	TG(2:0_16:0_18:0)	TG	108.1	14.3
514	DG(O-17:1_2:0)	DG	108.9	5.4
515	Cer(m18:2_16:0)	Cer	109.0	14.3
516	PC(39:7)	PC	109.3	7.4
517	PE(34:3)	PE	109.5	21.8
518	Cer(m12:0_16:0)	Cer	109.5	4.7
519	DG(O-16:1_18:1)	DG	109.5	1.5
520	PC(O-15:2_20:5)	PC	109.7	8.0
521	DG(P-4:0_17:0)	DG	109.8	11.8
522	Cer(d15:1_18:0)	Cer	110.0	6.1
523	Cer(m14:0_16:0)	Cer	110.4	6.3
524	TG(16:0_18:0)	TG	110.8	4.7
525	DG(14:0_16:0)	DG	111.3	1.7
526	TG(17:1_18:1_20:3)	TG	111.4	19.4
527	Cer(d18:1_16:0)	Cer	111.5	15.3
528	Cer(t14:1_18:3)	Cer	112.0	2.9
529	TG(15:0_16:0_16:0)	TG	112.0	26.5
530	TG(17:2CHO_4:0_4:0)	TG	112.3	15.7
531	DG(8:0_10:0)	DG	112.3	8.4
532	PC(O-31:2)	PC	112.6	8.7
533	SPH(d16:1)	SPH	112.6	5.0
534	PC(35:6)	PC	113.0	5.5
535	PE(O-34:2)	PE	113.0	1.4
536	PC(O-35:8)	PC	113.2	5.4
537	TG(O-7:0_3:0_8:0)	TG	113.2	7.6
538	PC(20:3_21:2COOH)	PC	113.4	6.3
539	Cer(d19:0_20:0)	Cer	113.4	3.7
540	PFAA(17:1)	PFAA	113.5	5.1
541	PC(41:9)	PC	113.6	4.8
542	DG(O-17:1_14:0)	DG	113.8	5.9
543	DG(18:0_20:5)	DG	114.0	29.0
544	PE(16:0_22:6)	PE	114.2	4.8
545	PC(34:2CHO)	PC	115.0	6.6
546	Cer(d15:1_11:0)	Cer	115.2	3.7
547	PC(22:5_18:0)	PC	115.3	1.8
548	PC(20:4_19:0)	PC	115.6	1.2
549	PC(19:1_15:2)	PC	115.8	8.0
550	Cer(d24:2_18:4)	Cer	115.9	6.2
551	PFAA(21:1)	PFAA	115.9	13.9
552	PE(18:1_22:6)	PE	116.1	5.9
553	Cer(d12:1_4:0)	Cer	116.4	24.6
554	Cer(t16:1_16:0)	Cer	116.5	8.3
555	TG(18:0_18:1)	TG	116.7	6.5
556	DG(O-17:1_16:0)	DG	116.9	5.0
557	Cer(d18:1_18:0)	Cer	117.0	12.8
558	PC(35:3COOH)	PC	117.0	17.2
559	DG(O-17:1_23:3)	DG	117.0	28.3

560	PC(4:1CHO_30:0)	PC	117.2	10.1
561	Cer(d15:1_16:0)	Cer	117.2	1.4
562	PFAA(15:1)	PFAA	117.6	3.7
563	TG(12:0_16:0_18:3)	TG	117.6	13.7
564	PC(O-41:11)	PC	117.9	5.3
565	PC(P-45:14)	PC	118.0	6.7
566	TG(O-18:2_18:0)	TG	118.5	7.5
567	PC(O-16:2_18:1)	PC	118.6	6.1
568	TG(18:2CHO_3:0_4:0)	TG	118.9	22.1
569	PFAA(22:1)	PFAA	118.9	4.4
570	SPH(d20:1)	SPH	119.4	8.5
571	PE(P-16:0_20:3)	PE	119.6	17.8
572	Cer(d16:0_18:3)	Cer	119.6	23.8
573	DG(19:1_16:0)	DG	120.2	9.7
574	PC(18:3_17:1)	PC	120.4	5.5
575	DG(P-5:0_17:0)	DG	120.6	20.9
576	PC(O-13:1_20:5)	PC	120.8	8.0
577	PE(P-16:0_20:5)	PE	121.1	8.2
578	PC(18:1_21:2COOH)	PC	121.4	5.8
579	Hex1Cer(d18:2_22:0)	Hex1Cer	121.6	16.3
580	PC(38:5CHO)	PC	121.7	9.0
581	Cer(d15:0_17:0)	Cer	121.9	20.5
582	DG(P-6:0_17:0)	DG	122.1	8.2
583	TG(3:0_4:0_18:2CHO)	TG	122.7	18.7
584	TG(12:0_14:0_18:1)	TG	122.7	10.1
585	PFAA(11:1)	PFAA	123.0	3.3
586	TG(P-6:0_15:0_2:0)	TG	123.1	4.5
587	PC(30:1COOH)	PC	123.5	12.4
588	PC(30:4COOH)	PC	124.0	14.0
589	PC(34:3)	PC	124.0	4.1
590	PC(20:5_19:1COOH)	PC	124.1	11.7
591	PC(15:2_20:4)	PC	124.5	13.4
592	PFAA(14:1)	PFAA	125.0	0.5

Table S15. The high-abundance lipids recovered in the lower phase following EMR-lipid processing.

No.	Lipid	Class	Abundance (10^6)	RSD (%)
1	DG(P-6:0_17:0)	DG	451.4	21.7
2	DG(P-6:0_17:0)	DG	451.4	21.7
3	DG(P-6:0_17:0)	DG	275.7	22.2
4	PFAA(22:1)	PFAA	249.5	24.1
5	PC(16:0_18:2)	PC	71.1	22.3
6	DG(P-6:0_17:0)	DG	41.9	1.7
7	DG(P-6:0_9:0)	DG	34.7	20.7
8	PC(16:0_18:1)	PC	24.3	24.1
9	PC(16:0_20:4)	PC	18.9	21.7
10	DG(P-6:0_9:0)	DG	15.7	20.6

11	PC(18:0_18:2)	PC	15.0	24.8
12	PFAA(22:2)	PFAA	13.3	5.5
13	PC(16:0_22:6)	PC	13.0	19.6
14	LPC(16:0)	LPC	12.4	5.8
15	PC(16:0_20:3)	PC	9.9	23.0
16	PFAA(18:0)	PFAA	8.3	10.9
17	PFAA(22:0)	PFAA	6.4	9.0
18	DG(P-6:0_13:0)	DG	5.9	2.3
19	TG(16:0_18:1_18:2)	TG	5.7	4.3
20	PFAA(20:1)	PFAA	5.6	0.6
21	DG(P-6:0_12:0)	DG	5.5	14.3
22	PFAA(24:1)	PFAA	5.4	6.6
23	DG(P-6:0_4:0)	DG	5.1	17.3
24	PC(18:0_20:4)	PC	5.0	23.7
25	PC(18:2_18:1)	PC	4.9	20.0
26	DG(P-6:0_9:0)	DG	4.4	18.9
27	DG(P-6:0_4:0)	DG	4.2	20.5
28	SM(d34:1)	SM	4.1	23.4
29	TG(16:0_18:1_18:1)	TG	3.3	12.9
30	TG(16:1_18:1_18:2)	TG	3.0	7.5
31	DG(P-6:0_17:0)	DG	2.6	10.8
32	PC(18:1_20:4)	PC	2.6	20.7
33	PFAA(16:0)	PFAA	2.5	12.3
34	DG(P-6:0_12:0)	DG	2.4	23.7
35	DG(P-6:0_12:0)	DG	2.3	3.2
36	PC(16:0_16:1)	PC	2.3	20.4
37	TG(16:0_16:1_18:1)	TG	2.2	2.5
38	MG(P-15:2)	MG	2.2	19.2
39	TG(12:1_3:0_8:0)	TG	2.1	0.6
40	DG(P-6:0_7:0)	DG	2.1	1.1
41	PC(18:0_22:6)	PC	2.0	21.7
42	PC(38:3)	PC	2.0	25.3
43	LPC(18:0)	LPC	1.9	8.0
44	DG(O-18:2_18:1)	DG	1.9	5.7
45	PC(18:2_18:2)	PC	1.8	28.6
46	PFAA(22:3)	PFAA	1.7	6.8
47	PC(34:3)	PC	1.7	26.1
48	PC(18:1_18:0)	PC	1.7	24.8
49	DG(P-6:0_17:0)	DG	1.6	13.2
50	PFAA(22:3)	PFAA	1.6	7.0
51	DG(P-5:0_17:0)	DG	1.5	20.9
52	Cer(d17:0_18:0)	Cer	1.4	19.9
53	SPH(d19:0)	SPH	1.3	29.4
54	WE(O-18:0_4:0)	WE	1.3	7.1
55	DG(P-6:0_17:0)	DG	1.3	10.3
56	DG(P-6:0_4:0)	DG	1.3	1.3
57	TG(3:0_6:0_14:1)	TG	1.3	1.2
58	PFAA(24:0)	PFAA	1.3	10.4
59	TG(P-5:0_16:1_2:0)	TG	1.3	0.6
60	TG(18:1_18:1_18:2)	TG	1.2	3.8

61	Cer(d17:0_16:0)	Cer	1.2	20.0
62	Cer(d19:0_16:0)	Cer	1.2	23.5
63	Cer(d17:0_16:0)	Cer	1.2	16.4
64	LPC(18:1)	LPC	1.1	10.2
65	DG(P-6:0_7:0)	DG	1.1	2.7
66	TG(16:0_16:0_18:1)	TG	1.1	4.8
67	LPC(18:2)	LPC	1.1	10.6
68	LPC(16:0)	LPC	1.1	10.0
69	TG(14:0_18:1_18:2)	TG	1.1	6.1
70	TG(15:1_8:0)	TG	1.0	2.6
71	PC(16:0_16:0)	PC	1.0	26.1

Table S16. The small metabolites detected in the upper phase of MTBE extracts.

No.	Metabolite	Abundance (10^6)	RSD (%)	Abundance ratio (%) [a]
1	L-(+)-Valine	3.9	27.1	6.0
2	decanoylcarnitine	1.1	19.3	6.5
3	Acetylcarnitine	16.6	23.5	6.6
4	9-Decenoylcarnitine	1.0	11.5	6.6
5	Uric Acid	2.4	23.0	7.9
6	propionylcarnitine	1.9	17.9	8.5
7	DL-Carnitine	66.5	28.1	9.1
8	2-methylbutyrylcarnitine	0.8	21.2	11.4
9	L-Proline	10.0	18.0	15.6
10	3-hydroxyoctanoylcarnitine	0.8	24.8	18.0
11	L-Hexanoylcarnitine	0.9	16.3	18.8
12	Hippuric acid	0.2	4.0	21.5
13	Creatine	17.6	4.5	22.4
14	L-(+)-Leucine	90.5	7.2	22.6
15	DL-Glutamine	0.8	18.2	26.8

[a] Abundance ratio is defined as the ratio between the abundance in the upper phase and the abundance in the lower phase.

Table S17. List of full names for non-lipid small metabolite classes.

No.	Major class	Major class abbreviation	Sub-class	Sub-class abbreviation
1	Alkaloids and derivatives	Alkaloids	Alkaloids and derivatives	Alk
2	Alkaloids and derivatives	Alkaloids	Morphinans	Mor
3	Benzenoids	Benzenoids	Indanes	Indane
4	Benzenoids	Benzenoids	Phenol esters	Phe
5	Benzenoids	Benzenoids	Benzene and substituted derivatives	Benzen
6	Benzenoids	Benzenoids	Pyrenes	Pyrene
7	Benzenoids	Benzenoids	Tetralins	Tetral
8	Exogenous	Exogenous	Exogenous metabolites	Exo

	metabolites			
9	Homogeneous non-metal compounds	Homogeneous non-metal	Non-metal oxoanionic compounds	Non
10	Homogeneous non-metal compounds	Homogeneous non-metal	Other non-metal organides	Oth
11	Hydrocarbons	Hydrocarbons	Unsaturated hydrocarbons	Uns
12	Lipids and lipid-like molecules	Lipids and lipid-like	Glycerolipids	GL
13	Lipids and lipid-like molecules	Lipids and lipid-like	Sphingolipids	SL
14	Lipids and lipid-like molecules	Lipids and lipid-like	Prenol lipids	PR
15	Lipids and lipid-like molecules	Lipids and lipid-like	Glycerophospholipids	GPL
16	Lipids and lipid-like molecules	Lipids and lipid-like	Steroids and steroid derivatives	Ste
17	Lipids and lipid-like molecules	Lipids and lipid-like	Fatty Acyls	FA
18	Nucleosides, nucleotides, and analogues	Nuc	Pyrimidine nucleosides	Pur
19	Nucleosides, nucleotides, and analogues	Nuc	Purine nucleosides	Pyrimi
20	Organic acids and derivatives	Organic acids	Organic carbonic acids and derivatives	OrgCar
21	Organic acids and derivatives	Organic acids	Peptidomimetics	Pep
22	Organic acids and derivatives	Organic acids	Vinylogous thioesters	Vin
23	Organic acids and derivatives	Organic acids	Carboximidic acids and derivatives	Carboxim
24	Organic acids and derivatives	Organic acids	Hydroxy acids and derivatives	Hyd
25	Organic acids and derivatives	Organic acids	Keto acids and derivatives	Ket
26	Organic acids and derivatives	Organic acids	Organic phosphonic acids and derivatives	OrgPho
27	Organic acids and derivatives	Organic acids	Organic sulfonic acids and derivatives	OrgSul
28	Organic acids and derivatives	Organic acids	Carboxylic acids and derivatives	Carboxyl
29	Organic nitrogen compounds	Organic nitrogen	Organonitrogen compounds	OrgNit
30	Organic oxygen compounds	Organic nitrogen	Organooxygen compounds	OrgOxy
31	Organohalogen compounds	Organohalogen	Halohydrins	Hal
32	Organoheterocyclic	Organoheterocyclic	Azoles	Azo

	compounds			
33	Organoheterocyclic compounds	Organoheterocyclic	Benzimidazoles	Benzim
34	Organoheterocyclic compounds	Organoheterocyclic	Benzothiazoles	Benzot
35	Organoheterocyclic compounds	Organoheterocyclic	Coumarans	Cou
36	Organoheterocyclic compounds	Organoheterocyclic	Dihydrothiophenes	Dihydrot
37	Organoheterocyclic compounds	Organoheterocyclic	Heteroaromatic compounds	Het
38	Organoheterocyclic compounds	Organoheterocyclic	Isoquinolines and derivatives	Iso
39	Organoheterocyclic compounds	Organoheterocyclic	Lactams	Lactam
40	Organoheterocyclic compounds	Organoheterocyclic	Lactones	Lacton
41	Organoheterocyclic compounds	Organoheterocyclic	Oxazinanes	Oxa
42	Organoheterocyclic compounds	Organoheterocyclic	Pyrrolidines	Pyrrol
43	Organoheterocyclic compounds	Organoheterocyclic	Tetrahydrofurans	Tetrah
44	Organoheterocyclic compounds	Organoheterocyclic	Trioxanes	Tri
45	Organoheterocyclic compounds	Organoheterocyclic	Dihydrofurans	Dihydrof
46	Organoheterocyclic compounds	Organoheterocyclic	Quinolines and derivatives	Qui
47	Organoheterocyclic compounds	Organoheterocyclic	Benzofurans	Benzof
48	Organoheterocyclic compounds	Organoheterocyclic	Diazines	Dia
49	Organoheterocyclic compounds	Organoheterocyclic	Piperidines	Pip
50	Organoheterocyclic compounds	Organoheterocyclic	Pyridines and derivatives	Pyridi
51	Organoheterocyclic compounds	Organoheterocyclic	Imidazopyrimidines	Imi
52	Organoheterocyclic compounds	Organoheterocyclic	Indoles and derivative	Indole
53	Organosulfur compounds	Organosulfur	Sulfoxides	Sul
54	Phenylpropanoids and polyketides	Phe Pol	Cinnamyl alcohols	Cinnanyl
55	Phenylpropanoids and polyketides	Phe Pol	Depsides and depsidones	Dep
56	Phenylpropanoids and polyketides	Phe Pol	Macrolactams	Mac
57	Phenylpropanoids	Phe Pol	Cinnamic acids and	Cinnamic

and polyketides	derivatives

Table S18. List of full names for lipid metabolite classes.

No.	Major class	Major class abbreviation	Sub-class	Sub-class abbreviation
1	Glycerolipids	GL	Diradylglycerols	DG
2	Glycerolipids	GL	Triradylglycerols	TG
3	Glycerolipids	GL	Monoradylglycerols	MG
4	Glycerophospholipids	GPL	Phosphatidylcholine	PC
5	Glycerophospholipids	GPL	Lysophosphatidylcholine	LPC
6	Glycerophospholipids	GPL	Phosphatidyl-ethanolamines	PE
7	Glycerophospholipids	GPL	Lyso-phosphatidyl-ethanolamines	LPE
8	Glycerophospholipids	GPL	Phosphatidylinositol	PI
9	Glycerophospholipids	GPL	Phosphatidylglycerol	PG
10	Glycerophospholipids	GPL	Phosphatidylserine	PS
11	Glycerophospholipids	GPL	Ceramide disaccharides	Hex2Cer
12	Sphingolipids	SL	Sphingomyelin	SM
13	Sphingolipids	SL	Ceramides	Cer
14	Sphingolipids	SL	Sphingosine phosphorylcholine	SPH
15	Sphingolipids	SL	Ceramide monosaccharides	Hex1Cer
16	Fatty Acyls	FA	Wax monoesters	WE
17	Fatty Acyls	FA	primary fatty acid amides	PFAA
18	Fatty Acyls	FA	N-acyl ethanolamines	AEA
19	Fatty Acyls	FA	Fatty acyl carnitines	AcCa

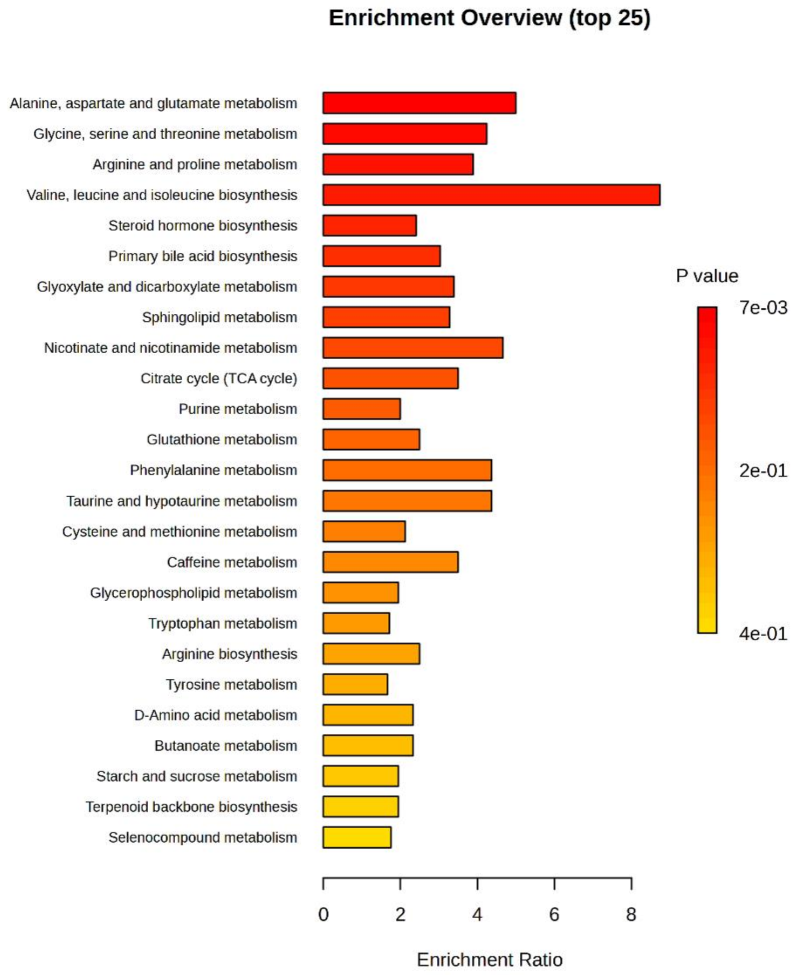

Figure S1. Pathway enrichment analysis of the annotated metabolites shared across maternal and cord blood sera.
